# Transcriptome profiling of differentially expressed genes in cytoplasmic male-sterile line and its fertility restorer line in pigeon pea (*Cajanus cajan* L.)

**DOI:** 10.1186/s12870-020-2284-y

**Published:** 2020-02-13

**Authors:** Swati Saxena, Sarika Sahu, Tanvi Kaila, Deepti Nigam, Pavan K. Chaduvla, A. R. Rao, Sandhya Sanand, N. K. Singh, Kishor Gaikwad

**Affiliations:** 1 ICAR-National Institute for Plant Biotechnology, New Delhi, 110012 India; 2 ICAR-Indian Agricultural Statistics Research Institute, New Delhi, 110012 India

**Keywords:** Cytoplasmic male-sterility, Differentially expressed genes, Illumina sequencing, Pigeon pea, Pollen, Transcriptome analysis

## Abstract

**Background:**

Pigeon pea (*Cajanus cajan* L.) is the sixth major legume crop widely cultivated in the Indian sub-continent, Africa, and South-east Asia. Cytoplasmic male-sterility (CMS) is the incompetence of flowering plants to produce viable pollens during anther development. CMS has been extensively utilized for commercial hybrid seeds production in pigeon pea. However, the molecular basis governing CMS in pigeon pea remains unclear and undetermined. In this study transcriptome analysis for exploring differentially expressed genes (DEGs) between cytoplasmic male-sterile line (AKCMS11) and its fertility restorer line (AKPR303) was performed using Illumina paired-end sequencing.

**Results:**

A total of 3167 DEGs were identified, of which 1432 were up-regulated and 1390 were down-regulated in AKCMS11 in comparison to AKPR303. By querying, all the 3167 DEGs against TAIR database, 34 pigeon pea homologous genes were identified, few involved in pollen development (*EMS1*, *MS1, ARF17*) and encoding MYB and bHLH transcription factors with lower expression in the sterile buds, implying their possible role in pollen sterility. Many of these DEGs implicated in carbon metabolism, tricarboxylic acid cycle (TCA), oxidative phosphorylation and elimination of reactive oxygen species (ROS) showed reduced expression in the AKCMS11 (sterile) buds.

**Conclusion:**

The comparative transcriptome findings suggest the potential role of these DEGs in pollen development or abortion, pointing towards their involvement in cytoplasmic male-sterility in pigeon pea. The candidate DEGs identified in this investigation will be highly significant for further research, as they could lend a comprehensive basis in unravelling the molecular mechanism governing CMS in pigeon pea.

## Background

Cytoplasmic male-sterility (CMS) is a maternally inherited trait in plants where they are unable to produce functional pollens [1]. It occurs due to unusual mitochondrial open reading frames (*orfs*) which are chimeric in structure and express proteins that restrict mitochondrial function and pollen development [2]. Cytoplasmic male-sterility phenotype is known to be restored by nuclear genes termed as the restorer of fertility (*Rf*) and hence result in the production of functional pollens in a plant with the aberrant mitochondrial genome [3]. CMS/*Rf* systems, therefore, give a clear view of the interaction and conflicts between nuclear and mitochondrial genome [4]. Cytoplasmic male-sterile systems along with the availability of restorer of fertility have emerged as a reliable tool for enhancing the yield of many crop plants by utilizing hybrid vigor or heterosis [5]. Hybrid seed production technology primarily involves three breeding systems: (a) CMS line, carrying male-sterile cytoplasm and devoid of functional nuclear *Rf* genes (b) Maintainer line, containing fertile cytoplasm along with similar *Rf* (c) Restorer line containing fertile cytoplasm along with functional dominant *Rf* gene(s). The F1 hybrids produced possess dominant *Rf* gene to restores the male-fertility and the blend of nuclear genes from the sterile and fertile lines results in hybrid vigor [2]. During the 1950s, the first CMS system used for hybrid corn production was maize CMS-Texas (CMS-T) system which substantially increased the hybrid seed production and remarkably enhanced the maize yield [6]. Till date, impressive advancement has been made in plants to comprehend the molecular basis of CMS and fertility restoration. A number of mitochondrial genes related to male-sterility have been reported such as *urf*13 from maize [7], *pcf* associated with *atp9* gene from petunia [8, 9], and the radish *atp8* of Ogura CMS [10]. However, in pigeon pea the gene associated with cytoplasmic male-sterility has not been much explored.

In flowering plants, anther development is a highly controlled mechanism which demands proper differentiation of the sporogenous tissue and timely programmed microsporogenesis [11]. The absence of male gametophyte (stamen) or defective development of anther, disruption of the pollen mother cells (PMC) or tapetum cells, abnormal development of pollen, and failure of anther dehiscence leads to pollen abortion [12, 13]. In all cases, CMS due to the dysfunctional mitochondrial genome is mainly responsible for the disruption of pollen development [14]. Previous studies on CMS provide definitive evidence that the organelle mitochondria are critical for the tapetum and microspores development [14]. During distinct stages of pollen development, the requirement of energy is extremely high, and even a slight defect in the mitochondrial function becomes deleterious and results in pollen sterility [15–17]. Previous studies have characterized many genes playing a pivotal role in anther development in *Arabidopsis* viz. *SPOROCYTELESS* (*SPL*)/*NOZZEL* (*NZZ*) [18–21], *EXCESS MICROSPOROCYTES 1* (*EMS1*) [22, 23], *TAPETUM DETERMINANT1* (*TPD1*) [24], *ABORTED MICROSPORES* (*AMS*) and *MALE STERILITY1* (*MS1*) [25–27], MYB gene family (*AtMYB103*) [28, 29], *RUPTURED POLLEN GRAIN1* (*RPG1*), *CYTOCHROME P450* (*CYP703A2*), *CYTOCHROME P450* (*CYP704B1*), acyl-coA synthetase5 (acos5), *LAP6* and *LAP5* [30–37] redundantly facilitate anther development and callose synthase5 (cals5) is essential for callose synthesis [38].

Pigeon pea (*Cajanus cajan* (L.) Millsp.) belongs to the family *Fabaceae* and regarded as the sixth major legume crop worldwide [39]. It is popularly known for its high protein content [40] and drought tolerance [41]. It is an important pulse crop worldwide and predominantly cultivated in the tropics and sub-tropics (http://faostat.fao.org). To meet the ever-growing demand for pigeon pea extensive research has been done during the last decade, a breakthrough has been accomplished in developing hybrid technology [42]. The pre-imperatives for large-scale hybrid seed production are pollen transfer method and advancement of stable cytoplasmic male-sterile (CMS) system. The male-sterility phenomenon could not be much explored in other legumes due to their self-pollinating nature. However, pigeon pea has a considerable natural out-crossing [43]. Till date, eight (A_1_ to A_8_) such CMS systems have been bred in pigeon pea by transferring the cytoplasm of the wild relatives in the nuclear background of the cultivated pigeon pea via interspecific-hybridization followed by several rounds of back-crossing [44]. Therefore, hybrid seed technology based on cytoplasmic male-sterility has given a chance to overcome the production constraints by efficiently increasing the yield potential in pigeon pea [45]. However, among the eight systems, A_2_ and A_4_ CMS system obtained from the wild-type *Cajanus scarabaeoides* and *Cajanus cajanifolius* respectively, are regarded as highly potential CMS systems [46–48]. The gene associated with CMS in A_4_ cytoplasm pigeon pea has been reported [49]. Recently, the genetics of fertility restoration in pigeon pea with A_2_ cytoplasm was studied in detailed [47]. Even though A_2_ cytoplasm has played a significant role in hybrid seed production, yet the underlying molecular processes governing CMS in A_2_ cytoplasm pigeon pea is still not known.

Presently, the next generation sequencing technology has revolutionized the field of life sciences and offers extraordinary speed and cost-effectiveness to study genomic and transcriptome data [50, 51]. RNA-Seq has emerged as an effective approach for transcriptome profiling which provides accurate measurement of gene expression level [52]. Transcriptome sequencing offers ease of identification and evaluation of thousands of genes in a single analysis [53]. Transcriptome analysis of the cytoplasmic male-sterile lines has been previously reported in many crops such as radish [54], watermelon [55], soybean [56], tomato [57], *Brassica napus* [58], chili pepper [59],cotton [60–62], sweet orange [63] leading to identification of several candidate genes in these systems. In this study, comparative transcriptome analysis of an A_2_ CMS system derived cytoplasmic male-sterile, and its fertility restorer line in pigeon pea was performed primarily to detect differentially expressed genes participating in pollen development which might help in understanding the CMS mechanism in this crop.

## Results

### Pollen fertility analysis and phenotypic characterization of sterile and fertile buds

**Fig. 1:**
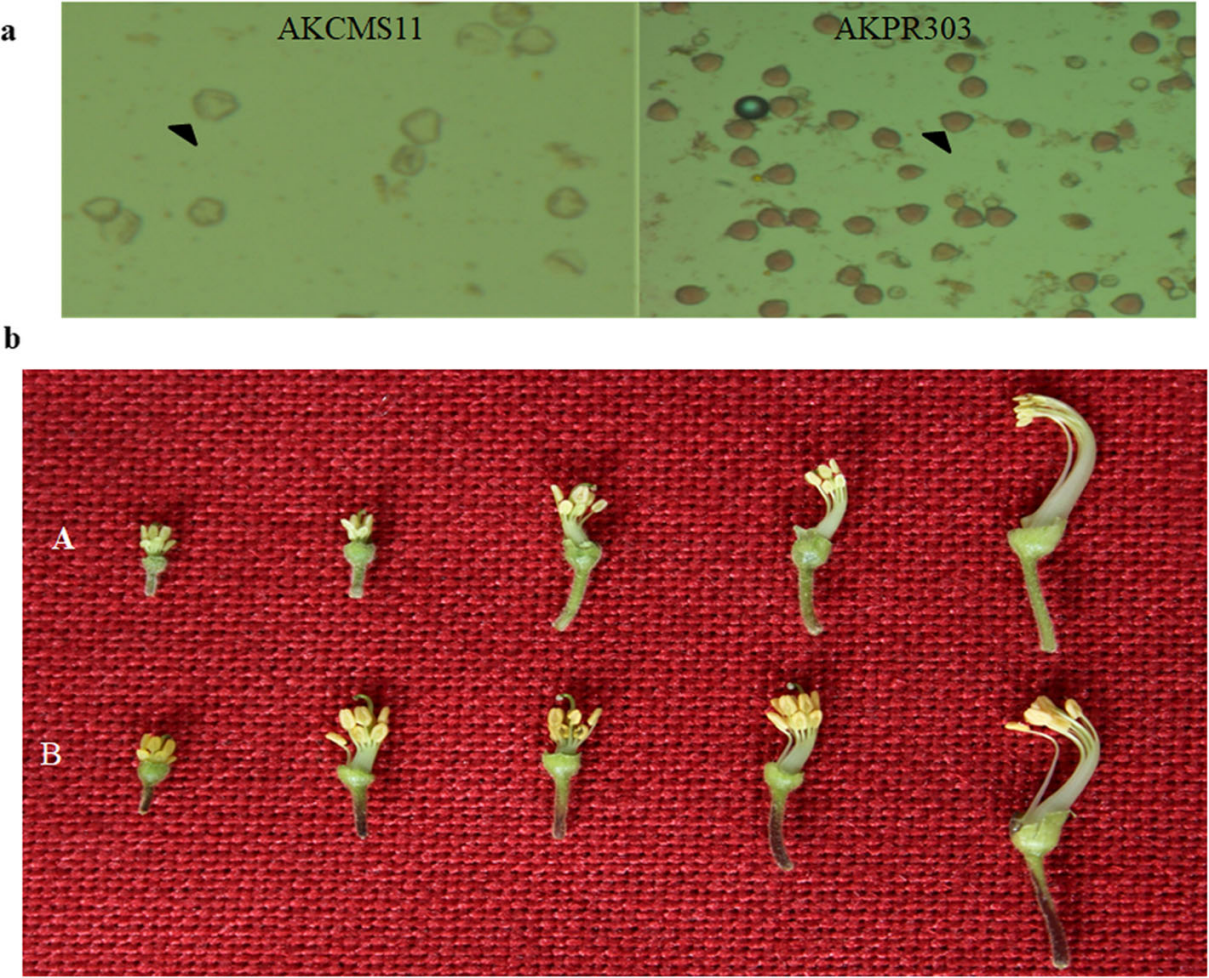
**a** Acetocarmine staining differentiating the pollens of AKCMS 11 (sterile line) and AKPR303 (fertile line). Fertile pollens are darkly stained whereas sterile pollens are almost colorless. **b** Phenotypic characterization of the sterile and fertile floral buds. **A**: Phenotype of sterile AKCMS11 buds; **B**: Phenotype of fertile AKPR303 buds

Pollens from male-sterile and fertile buds were observed under a microscope and were evaluated to determine fertility. Upon observation it was seen that the pollens grains showing red acetocarmine staining were fertile while sterile pollens remained unstained (Fig. 1a). During the phenotypic investigation of the sterile (AKCMS 11) and fertile (AKPR303) flower buds we observed that the filaments and anthers of the sterile flowers were smaller in contrast to the fertile flowers. It was clearly visible that the anthers of the fertile line were denser than the sterile line (Fig. 1b).

### Transcriptome sequencing and de novo assembly

**Fig. 2:**
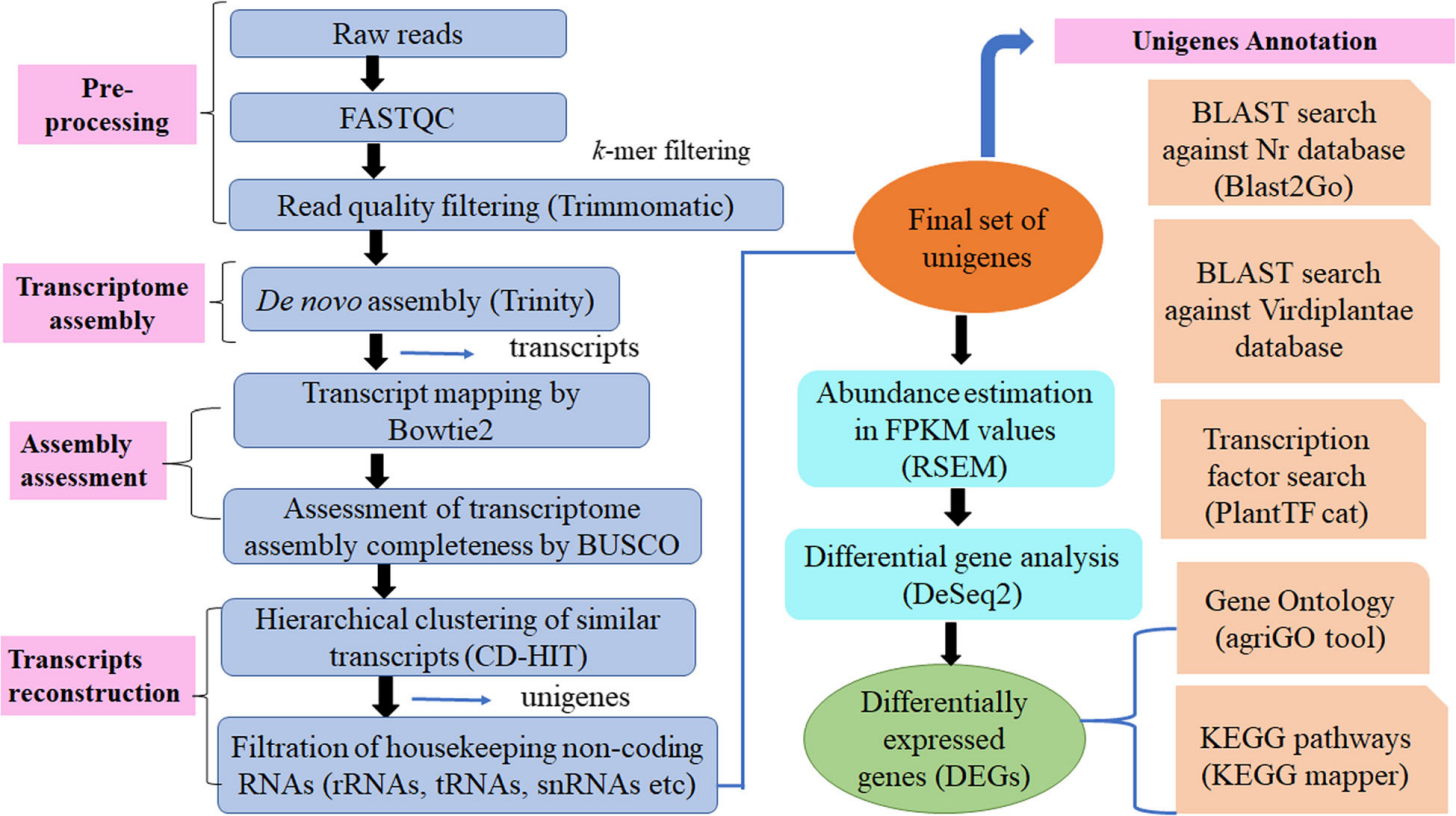
Systemic representation showing workflow of the analysis done

**Fig. 3:**
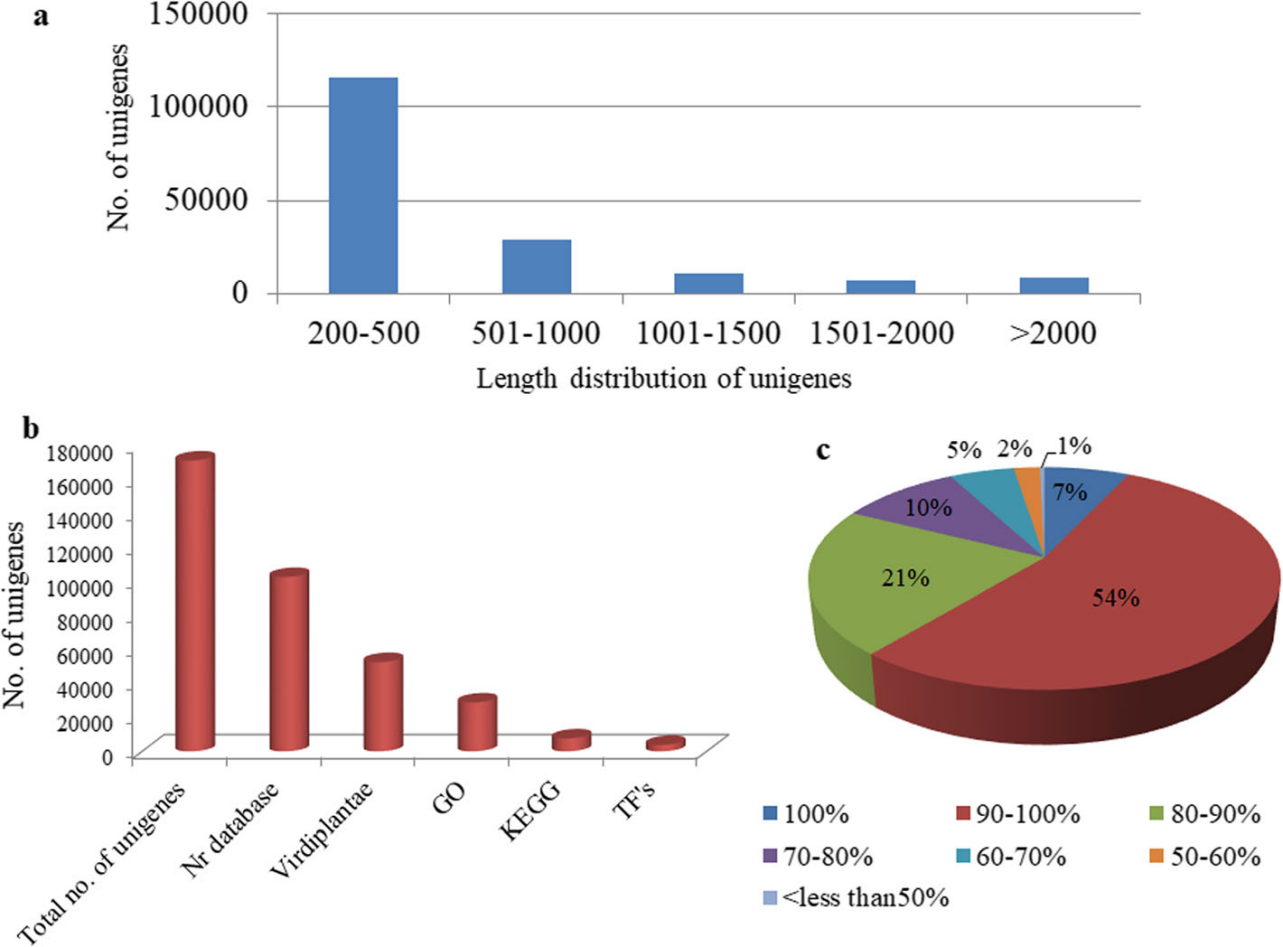
**a** Length distribution of the assembled unigenes. **b** Homology search and annotation statistics of pigeon pea unigenes. **c)** Similarity distribution of the BLASTX hits for each unigenes against nr database

To create a reference transcriptome, we prepared cDNA libraries from the buds of male-sterile (CMS) and fertile (restorer) lines in pigeon pea (two replicates each) and subjected to Illumina paired-end sequencing. Workflow diagram of the analysis done is shown in Fig. 2. High throughput sequencing run generated a total of 109,600,365 and 72,575,988 raw paired-end reads of ~ 100 bp in the sterile and fertile buds, respectively. Also, the correlation between the replicate datasets of the sterile AKCMS11 and fertile restorer AKPR303 was analyzed by calculating Pearson’s correlation coefficient (*r* < 0.9) using an R studio package. Correlation analysis indicated that the replicate datasets of the sterile and fertile lines were positively correlated with Pearson’s coefficient value as r = 0.72 and r = 0.74, respectively (Additional file 1: Figure S1). The per base sequence quality and *k*-mer content of the raw paired-end reads was also determined (Additional file 1: Figure S2 and Table S1). After adapter trimming and removal of poor quality reads, 101,676,579 (49.5 Gb) and 69,481,226 (32.7 Gb) clean reads were attained for AKCMS11 (sterile) and AKPR303 (fertile) lines, respectively. Trinity software was further employed for de novo assembly of the pooled reads (171,157,805) from both the samples. There was a total of 1,98,587 transcripts with an average length of 641.34 bp, and the N50 value of 1009 bp (L50 = 6 contigs). We utilized Bowtie2 to align the clean reads to the assembled transcriptome, alignment score of 92.84 and 92.84% was obtained for sterile AKCMS11 (replicate 1 and replicate 2), respectively. Similarly, alignment score of 92.61 and 92.05% was obtained for fertile AKPR303 (replicate 1 and replicate 2), respectively. (Additional file 1: Table S2). An overall alignment rate of 92.58% was obtained indicative of the robust assembly. BUSCO analysis revealed that our assembly was relatively complete, with 98% (*n* = 297) of BUSCOs detected as complete sequences, just 2% (*n* = 6) as fragmented sequences, and none were missing in the assembly with eukaryotic lineage. Similarly, 93.5% (*n* = 402) of BUSCOs detected as complete sequences, 5.8% (*n* = 25) as fragmented sequences, and just 0.7% (*n* = 3) were missing in the assembly with viridiplantae lineage (Table 1). After the de novo assembly, 1,72,061 unigenes were obtained by using CD-HIT software. These unigenes were further BLASTN searched against the Rfam database to remove the non-coding RNAs (rRNA, tRNA, snoRNAs, snRNA etc.). Finally, a set of 1,71,095 unigenes were obtained with an average length and N50 of 561.66 bp and 757 bp, respectively (Table 2). The shortest and longest unigenes identified were 201 bp and 16,008 bp, respectively. We obtained 1,15,792 unigenes (67.7%) with a length range from 200 to 500 bp. 17,692 unigenes (10.3%) were greater than 1000 bp and 8700 (5.1%) were greater than 2000 bp (Fig. 3a). The average GC content of the unigenes was 45.8%. The RNA-Seq raw data generated from this investigation has been deposited in the NCBI Sequence Read Achieve (SRA) database (SRX3740150, SRX3740151, SRX3740153, SRX3740152) for sterile and fertile buds, respectively.

**Table 1:** BUSCO completeness assessment conducted by using eukaryotic ortholog database (eukaryota_orthoDB9) and viridiplantae ortholog database (viridiplantae_orthoDB10)

BUSCOs (Number of BUSCO units found)		
	eukaryotic ortholog database	viridiplantae ortholog database
Complete BUSCOs	297	402
Complete and Single-Copy BUSCOs	186	315
Complete and duplicated BUSCOs	111	87
Fragmented BUSCOs	6	25
Missing BUSCOs	0	3
Total BUSCOs searched	303	430

**Table 2:** Summary of Illumina sequencing and de novo transcriptome

Read processing			
	AKCMS11 (two replicates)	AKPR303 (two replicates)	Total
Raw reads	109,600,365	72,575,988	182,176,353
Clean reads (after trimming)	101,676,579	69,481,226	171,157,805
Average read length (bp)	100	100	˗
Trinity based de novo assembly			
No. of transcripts (n)	1,98,587		
Percentage GC	41.14		
Contig N10 (bp)	3148		
Contig N20 (bp)	2300		
Contig N30 (bp)	1788		
Contig N40 (bp)	1375		
Contig N50 (bp)	1009		
L50 value	6		
Median contig length	357		
Average length	641.34		
Total assembled bases	127,362,415		
**Unigenes**			
Total number of unigenes (n)	1,71,095		
Average length (bp)	561.66		
N50 length (bp)	757		
Total assembled bases	97,193,132

### Functional annotation and classification of the unigenes

Annotation results showed that, 1,02,463 (60%), 52,639 (30.6%), 28,812 (16.7%) and 7653 (4.4%) unigenes annotated in Nr, Virdiplantae, GO and KEGG databases, respectively (Fig. 3b). In total, 1,02,463 (60%) unigenes were annotated in the nr database, while the remaining 40% unigenes did not show any sequence similarity to the nr database (Additional file 2: Table S1). Sequence similarity distribution revealed that 61% of the aligned unigenes had sequence similarity more than 90%, on the other hand, 38.6% unigenes had similarity value between 50 to 90% and only 0.4% unigenes had lower than 50% (Fig. 3c). A total of 52,639 unigenes were uniquely mapped to the Virdiplantae database (Additional file 2: Table S2).

A total of 28,812 unigenes were assigned GO terms and were distributed into 53 GO categories including 26 biological processes, 14 molecular functions, and 13 cellular components. In the biological process category, “cellular process” (19,472 unigenes) was the most predominant functional group followed by “metabolic process” (17,365 unigenes) and “biological regulation” (9627 unigenes). Among the molecular function category, “binding” (19,531 unigenes), “catalytic activity” (14,757 unigenes) and “transcription regulator activity” were the main functional groups. In regard to the cellular component, “cell” (22,732 unigenes) and “cell part” (22,732 unigenes) were the largest categories followed by “organelle” (15,348 unigenes) and “macromolecular complex” respectively (Additional file 2: Table S3 and Additional file 3: Figure S1).

The unigenes were mapped to the KEGG pathway database, 7653 unigenes were assigned KEGG orthologos number involving 132 pathways. The result demonstrated that the five largest pathways were “metabolic pathways” (ko01100), “biosynthesis of secondary metabolites” (ko01110), “plant hormone signal transduction (ko04075), “carbon metabolism” (ko01200) and “biosynthesis of amino acids” (ko01230). The 7653 unigenes were allocated into six functional categories (Additional file 2: Table S4 and Additional file 3: Figure S2). A total of 5499 unigenes were found in “metabolism” with 1363 (24.8%) unigenes involved in “metabolic pathways”, 881 (16%) in “biosynthesis of secondary metabolites”, 870 (15.8%) in “carbohydrate metabolism”, 665 (12.1%) in “amino acid metabolism”, 458 (8.3%) in “lipid metabolism” and other sub-categories. 1222 unigenes accounted for the category of “genetic information and processing” in which 449 (36.7%) unigenes were engaged in “folding, sorting and degradation” followed by “translation” 446 (36.5%), “transcription” 181 (14.8%) and “replication and repair” 146 (11.9%).“Environmental information processing” was represented by a total of 462 unigenes with 416 (90%) involved in “signal transduction” and 46 (10%) in “membrane transport”. Additionally, 300 and 155 unigenes were classified in “cellular processes” and “organismal systems” categories, respectively.

### Identification of transcription factors

**Fig. 4:**
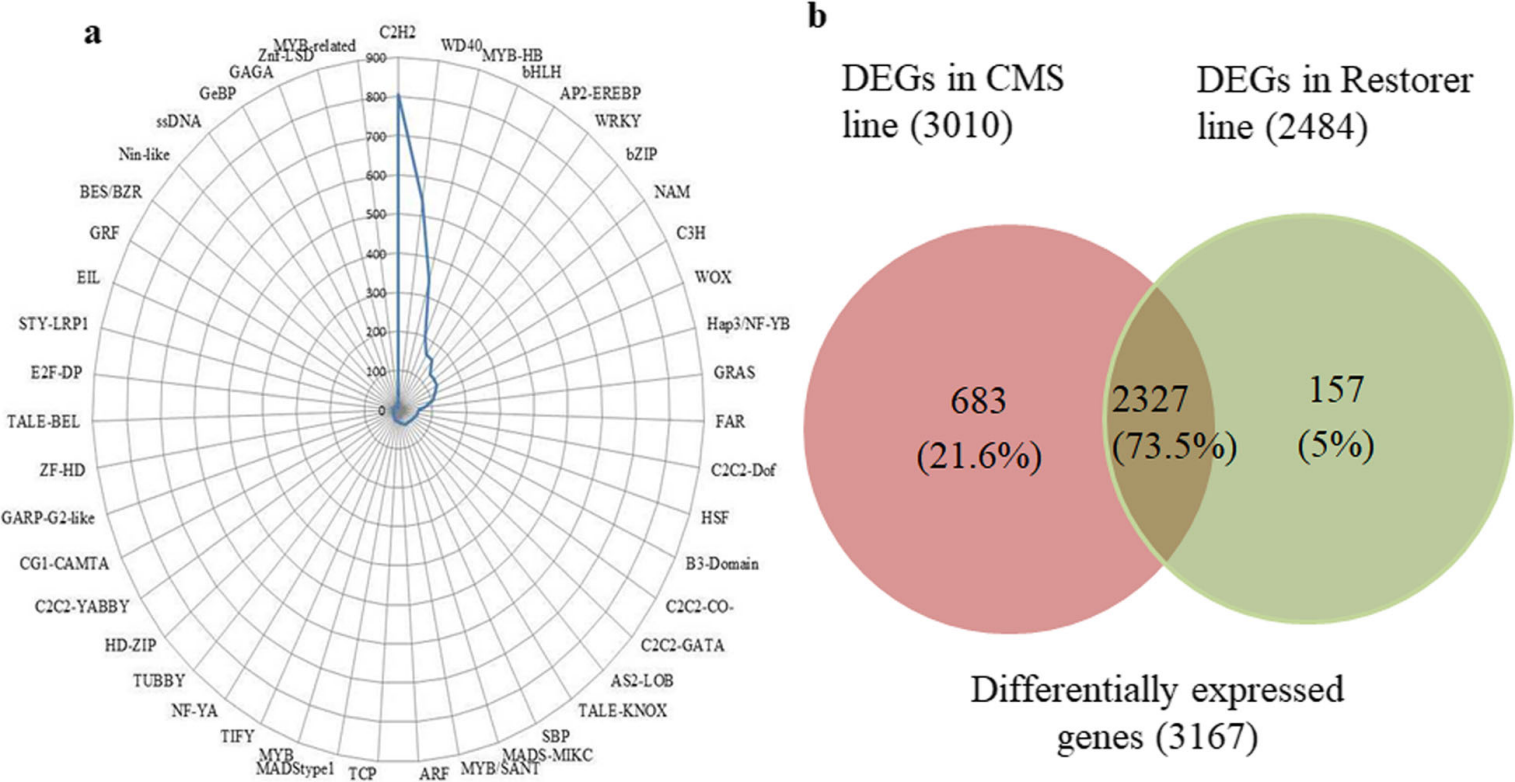
**a** Transcription factors identified in the transcriptome data of *Cajanus cajan.* **b** Number of differentially expressed genes (DEGs) in sterile AKCMS11 and restorer AKPR303

PlantTFcat online tool [64] was utilized in the present study, for identification of transcription factors in pigeon pea. A total of 3803 unigenes (2.2% of the transcriptome) (Fig. 3b) were cataloged into 52 putative transcription factors (TF) families. Amid the 52 TF family, “C2H2” was found to be the most predominant category with 806 (21.2%) unigenes followed by “WD40-like” (540 unigenes, 14.2%), “MYB-HB-like” (344 unigenes, 9%), “bHLH” (200 unigenes, 5.3%), “AP2-EREBP” (163 unigenes, 4.3%), “WRKY” (161 unigenes, 4.2%) and “bZIP” (129 unigenes, 3.4%) transcription factors (Fig. 4a and Additional file 2: Table S5).

### Identification of differentially expressed genes between lines AKCMS11 and AKPR303

We identified 3167 differentially expressed genes (DEGs) between the two lines containing 1432 up-regulated and 1390 down-regulated in the AKCMS11 sterile buds as compared to AKPR303 fertile buds (Additional file 2: Table S6). Additionally, 683 DEGs were uniquely expressed in the sterile genotype and 157 DEGs were distinctively expressed in the fertile restorer (Fig. 4b).

### GO and KEGG enrichment analysis of the DEGs

**Fig. 5:**
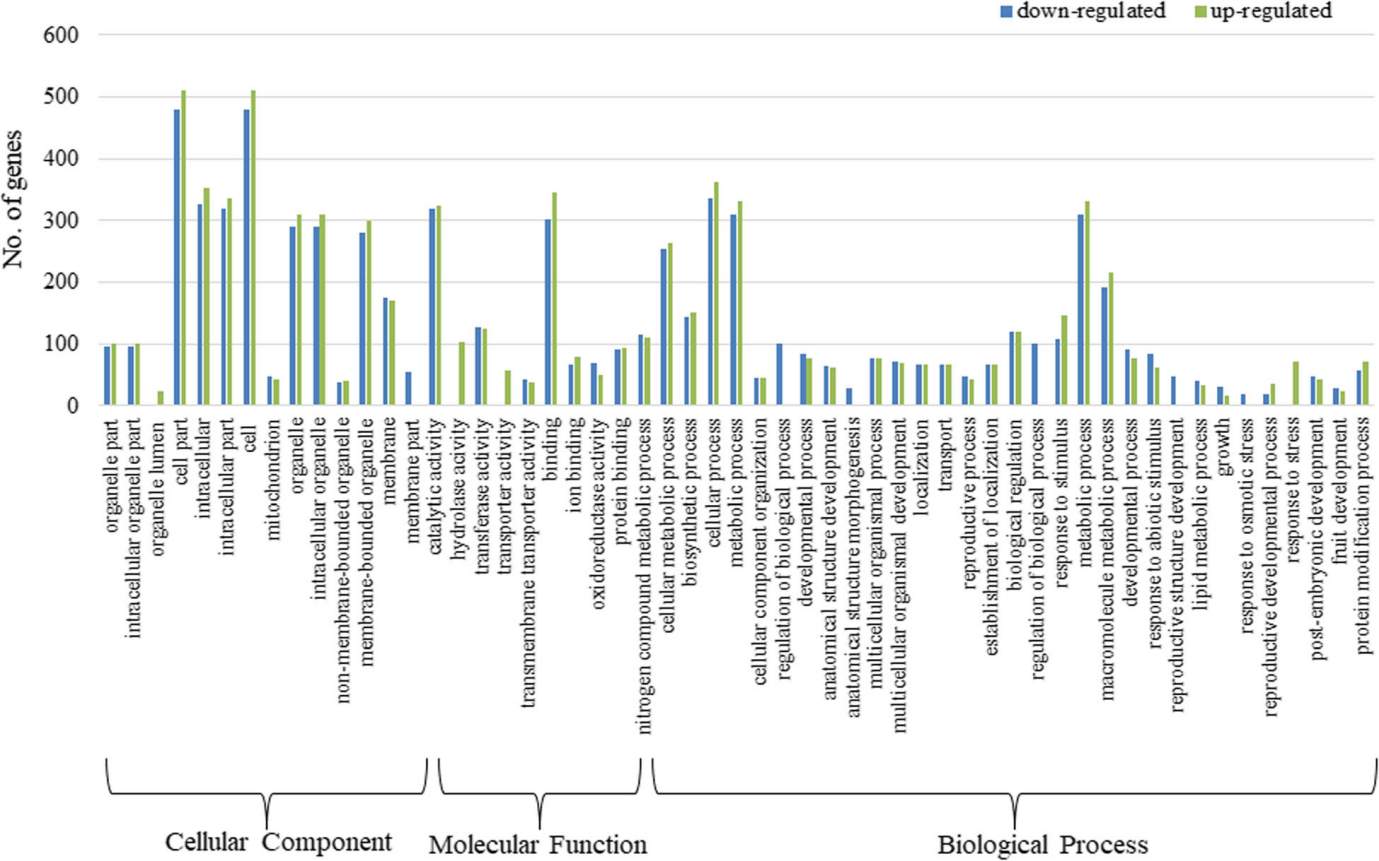
GO enrichment analysis of the DEGs. X-axis represents the sub-categories; Y-axis represents number of genes in each sub-category. Blue and green indicates down-regulated and up-regulated genes in a sub-category

**Fig. 6:**
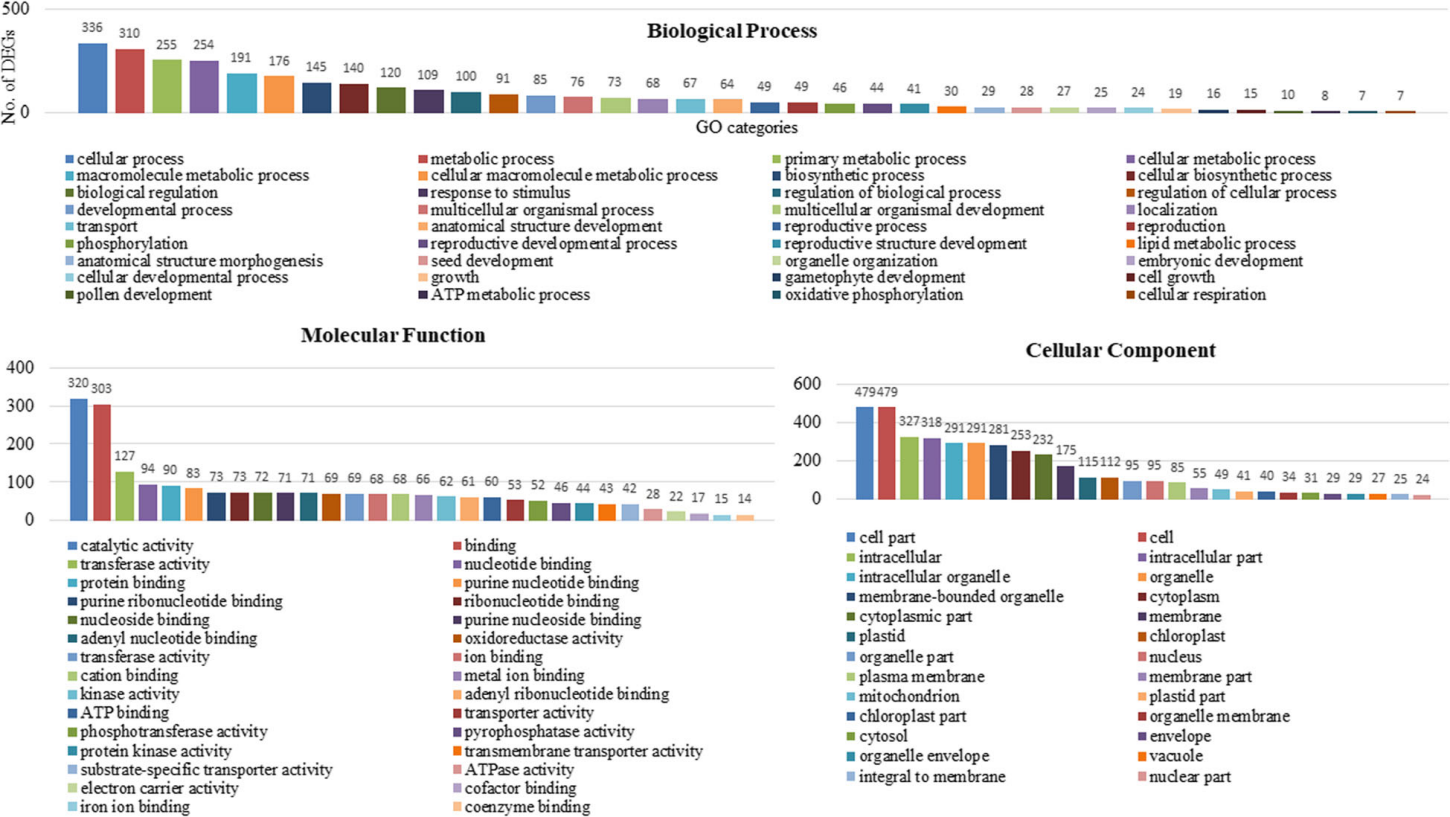
Significantly over-represented GO terms in down-regulated DEGs

**Fig. 7:**
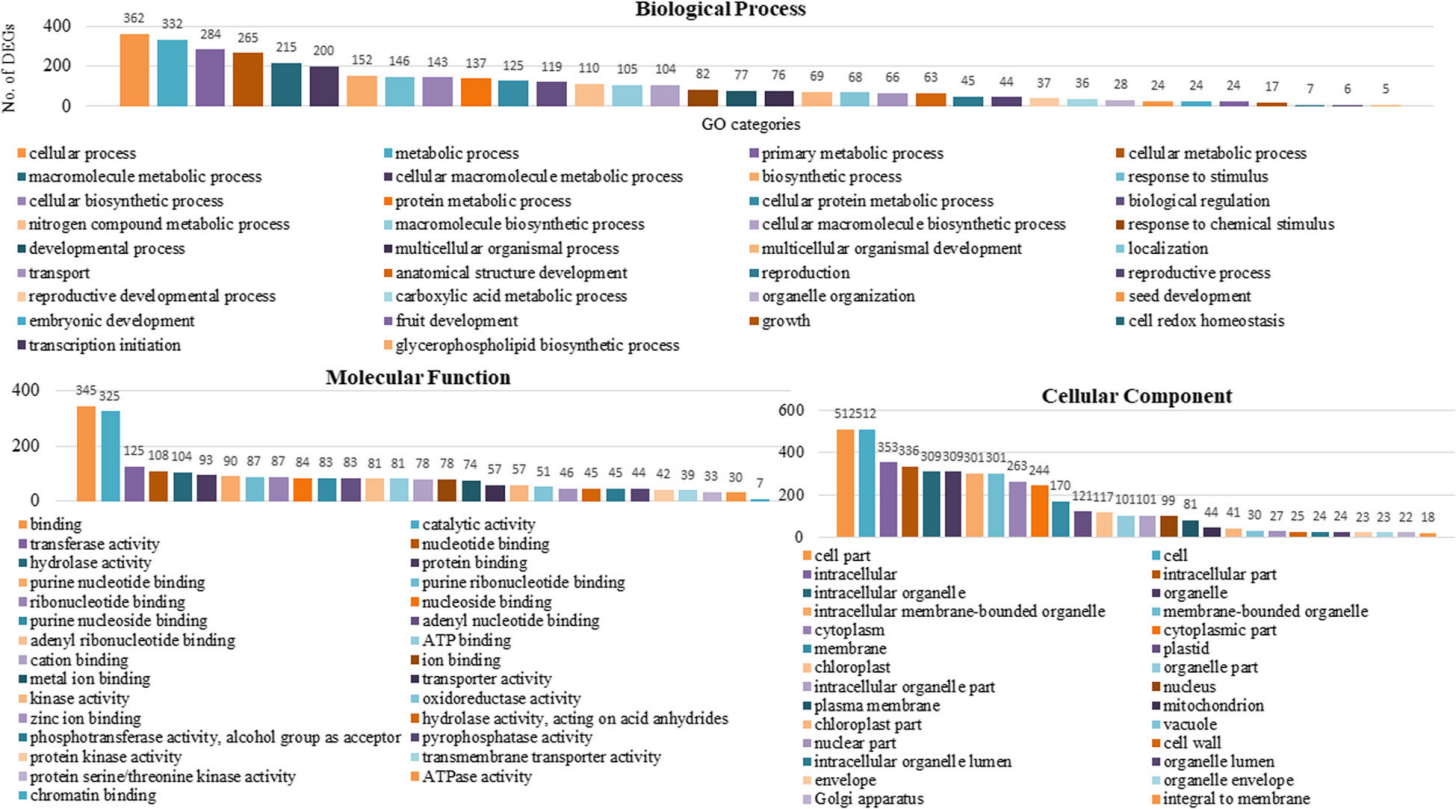
Significantly over-represented GO terms in up-regulated DEGs

To gain insight into the putative functions of the DEGs, we examined the significantly enriched Gene Ontology (GO) terms in DEGS. The annotated DEGs were assigned to 55 major groups covering three main ontologies: biological process, cellular component and molecular function (Fig. 5). The identified GO terms were further classified into down-regulated and up-regulated groups. In the down-regulated DEGs, a total of 221 GO terms were assigned, including 121, 59 and 41 GO terms in “biological process”, “molecular functions” and “cellular component”, respectively (Additional file 2: Table S7). In up-regulated DEGs, 174 GO terms were assigned, including 78 “biological process”, 53 “molecular functions” and 43“cellular component” (Additional file 2: Table S8). Overall, among the biological process category, the significantly over-represented GO terms were “cellular process”, followed by “metabolic process”, “primary metabolic process” and “cellular metabolic process”. “Catalytic activity”, “binding” and “transferase activity” were the most significantly expressed GO terms in the molecular functions category. Among the cellular component category, “cell part”, “cell” and “intracellular component” were the most significant GO terms (Figs. 6 and 7). Interestingly, many GO terms were specific to down-regulated DEGs and were involved in “pollen development” (10 DEGs), “reproductive structure development” (41 DEGs), “gametophyte development” (16 DEGs), and “oxidative phosphorylation” (7 DEGs), suggesting these may be related to male-sterility in AKCMS11. Hierarchical tree graph of over-represented GO terms in the biological process category of down-regulated and up-regulated DEGs are provided in Additional file 3: Figure S3 and S4.

**Fig. 8:**
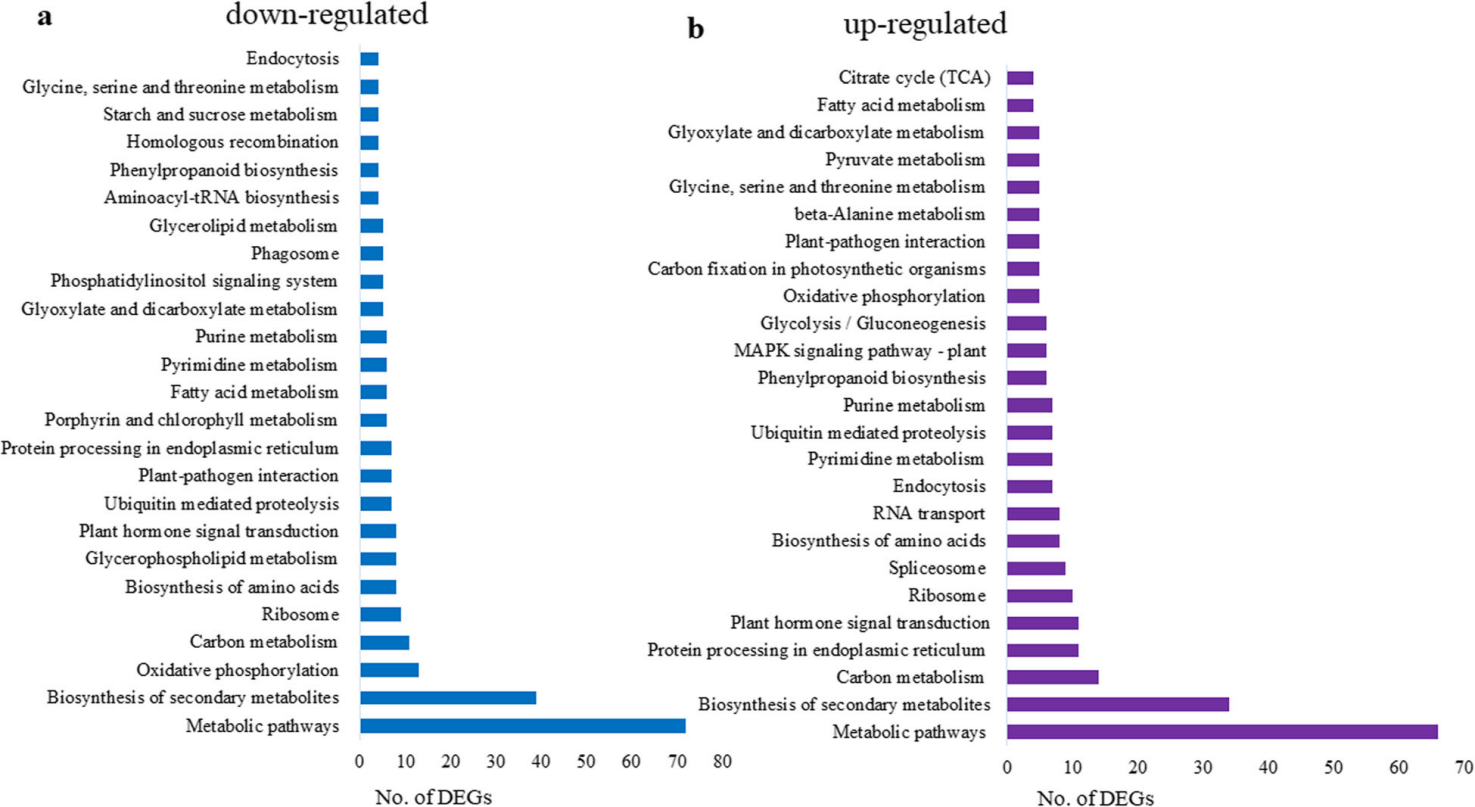
Top 25 significantly enriched KEGG pathways. **a** For down-regulated DEGs. **b** For up-regulated DEGs

KEGG pathway enrichment analysis was performed to discern the metabolic pathways in which the DEGs were involved using the KEGG pathway database. In total, 110 enriched pathways were identified, of which 77 pathways were significantly enriched in both down and up-regulated DEGs whereas 15 and 19 pathways were specific to down and up-regulated DEGs, respectively. A total of 377 down-regulated and 396 up-regulated DEGs were assigned to 92 and 96 KEGG pathways, respectively (Additional file 2: Table S9). The top 25 significantly enriched pathways for down and up-regulated DEGs are mentioned in Fig. 8. The down-regulated DEGs were significantly over-represented in “metabolic pathways” (72 genes), “biosynthesis of secondary metabolites” (39 genes), “oxidative phosphorylation” (13 genes), “carbon metabolism” (11 genes), “ribosome” (9 genes) and glycerophospholipid metabolism (8 genes). Consistent with the GO analysis “oxidative phosphorylation was enriched with down-regulated DEGs. Several down-regulated DEGs participated in starch and sucrose metabolism, glycolysis, pentose phosphate pathway, reactive oxygen species (ROS) generation/scavenging and alpha-linolenic acid metabolism. Among the up-regulated DEGs, the most predominant pathway was “metabolic pathways” (66 genes) followed by “biosynthesis of secondary metabolites” (34 genes), “carbon metabolism” (14 genes), “protein processing in endoplasmic reticulum” (11 genes) and “plant hormone signal transduction” (11 genes). Also, some up-regulated genes were involved in “ascorbate and aldarate metabolism”, “linolenic acid metabolism” and “photosynthesis” etc. (Additional file 2: Table S9).

**Fig. 9:**
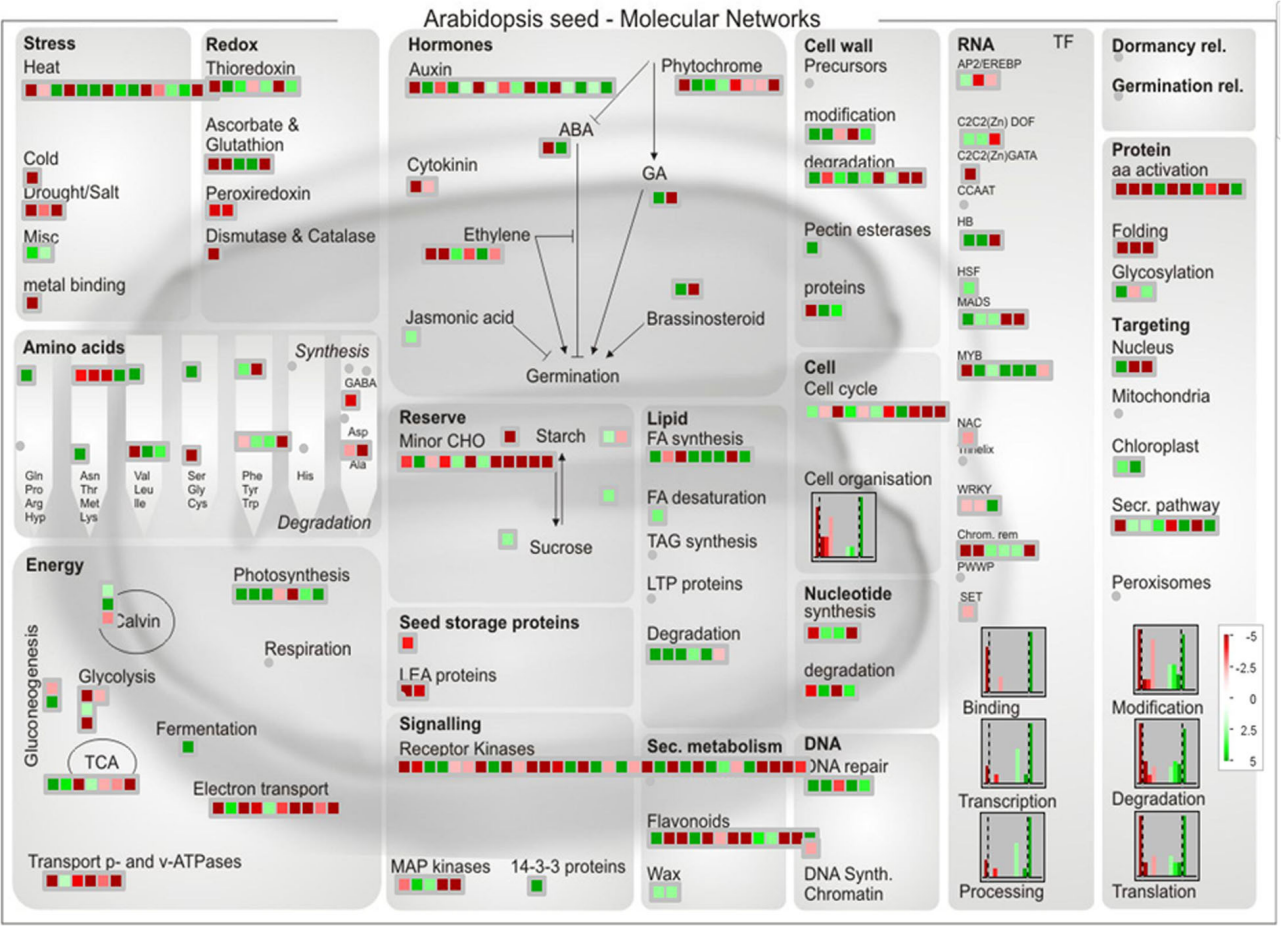
Overview of differentially expressed genes (DEGs) involved in diverse metabolic pathways. DEGs were selected for the metabolic pathways using MapMan software. The colored red and green boxes indicate down-regulated and up-regulated genes, respectively. The scale bar represent fold change values

Additionally, MapMan tool [65] was also used to visualize significant male-sterility related DEGs involved in different metabolic pathways. All the 3167 differentially expressed genes between sterile AKCMS11 and fertile AKPR303 were annotated to the TAIR database (http://www.arabidopsis.org) and finally, 951 DEGs were identified to be homologs of 930 Arabidopsis genes (Additional file 2: Table S10). To further explore, the potential functions of these DEGs in male-sterility, the Arabidopsis homologs genes were studied in MapMan to identify different metabolic processes in which these are involved (Fig. 9). In our network, the most significant differentially expressed genes were related to Energy/ATP synthesis, ROS metabolism, hormones, secondary metabolism and cell cycle. The expression of genes involved in ‘Energy (Glycolysis, TCA cycle, electron transport chain, transport p- and v-ATPase’s) was down-regulated in the sterile (AKCMS11) buds. Moreover, the genes related to ‘Redox’ (Ascorbate & Glutathione, Peroxiredoxin, Dismutase & Catalase) and cell cycle were also down-regulated in the sterile buds. Additionally, the genes implicated in ‘Auxin & Jasmonic acid synthesis’ were up-regulated in sterile buds, while the ones related to ‘Signaling pathway’ were down-regulated. Also, the expression of genes involved in ‘Lipid’ (Degradation & FA synthesis) and ‘Cell wall’ (degradation & modification) were mostly up-regulated, whereas genes related to ‘Secondary metabolism’ (Flavonoids) were down-regulated in the sterile buds compared to fertile buds. Among the transcription factors, NAC, WRKY (except one), CCAAT, SET, C2C2(Zn) GATA and AP2/EREBP (except one) were down-regulated in the sterile buds, whereas the majority of the MYB, MADS and C2C2(Zn) Dof transcription factors were up-regulated in comparison to the fertile buds.

### Candidate DEGs associated with male-sterility

**Fig. 10:**
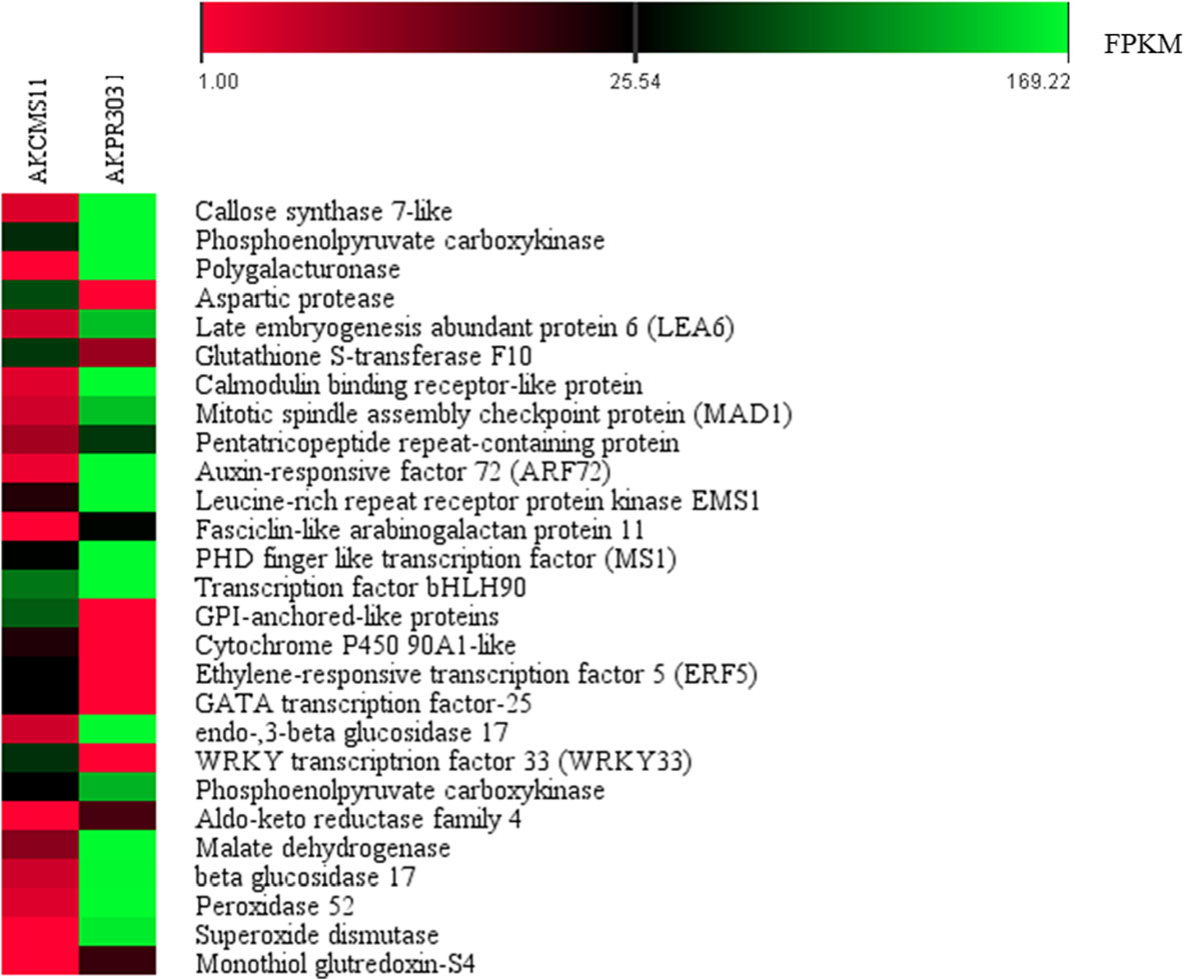
Heat map of expression profiles of some of the focused genes related to cytoplasmic male-sterility. Color from red to green, indicated that the FPKM values were small to large, red color indicates low level of gene expression and green color indicates high level of gene expression

The bioinformatics analysis gave us a deeper insight into the differentially expressed genes (DEGs) between the sterile (AKCMS11) and fertile genotypes (AKPR303). In this present investigation, several subsets of differentially expressed genes were identified which are potentially related to pollen development (Fig. 10).

### Putative gene related to pollen development

In flowering plants, pollen development is a complicated and sequential proces*s* of sexual reproduction. *Arabidopsis thaliana* is considered as the model plant to study putative genes related to pollen development [58]. To understand the mechanism related to male sterility, we queried all the 3167 DEGs in the TAIR database (http://www.arabidopsis.org). Additionally, all the 3167 DEGs were further compared with the *Arabidopsis* database (http://www.arabidopsis.org) to identify homologs using OrthoDB v10 [66] (Additional File 2: Table S11). In our study, 34 DEGs were found to be homologs of *Arabidopsis* genes associated with the development of pollen (Table 3). We found 10 DEGs involved in regulation of pollen development, among which 1 DEG encoding *EXCESS MICROSPOROCYTES 1* (*EMS1*), 1 DEG encoding PHD-finger transcription factor *MALE STERTILITY1* (*MS1*), 2 DEGs encoding callose synthase 7, 1 DEG encoding *AUXIN-RESPONSIVE FACTOR* (*ARF17*), 4 DEGs encoding *CYTOCHROME P450-like*, and 1 DEGs encoding aspartic protease. 5 DEGs were up-regulated and 5 were down-regulated in the sterile AKCMS11 in comparison to fertile AKPR303. 3 DEGs encoding polygalacturonase (PG) and endo-1,3- beta-glucosidase-like were involved in pollen cell wall remodeling. Total 5 DEGs encoding arabinogalactan glycoproteins (AGPs) and GPI-anchored like protein were involved in pollen tube growth. Single stress induced DEG was present, encoding late embryogenesis abundant protein (LEA) showing significant down-regulation in AKCMS11 with respect to AKPR303. We identified, 3 DEGs involved in the cell division process. Additionally, 12 DEGs with related functions were also identified in this study (Table 3).

**Table 3:** Pigeon pea differentially expressed genes (DEGs) homologous to *Arabidopsis* male-sterility/reproduction genes

^a^Query ID	^b^Log_2_FC	^c^Subject Id	E-value	Score	Symbol	Gene annotation
**Down-regulated**						
*Ccajan*_41968_c0_g1_i1	−6.21	AT3G12830.1	1.68E-10	55.8	ARF72	auxin-responsive factor 72 (*ARF72*)
*Ccajan*_10186_c0_g1_i1	−4.53	AT5G07280.1	7.10E-21	86.3	EMS1	leucine-rich repeat receptor protein kinase *EMS1*
*Ccajan*_37653_c0_g4_i5	−9.15	AT5G60490.1	7.72E-05	32	FLA11	Fasciclin-like arabinogalactan protein 11
*Ccajan*_38216_c1_g2_i3	−5	AT1G54860.1	1.32E-35	128		GPI-anchored-like proteins
*Ccajan*_34303_c0_g2_i2	−4.27	AT1G77250.1	2.17E-33	120		PHD finger like transcription factor (*MS1*)
*Ccajan*_44775_c0_g1_i1	−5.45	AT1G06490.2	2.42E-04	38.5	CALS7	callose synthase 7
*Ccajan*_52234_c0_g1_i1	−6.38	AT3G14570.2	0	1100	CALS8	callose synthase 8
*Ccajan*_83966_c0_g1_i1	−8.85	AT1G02790.1	2.75E-98	303	PGA3	Polygalacturonase (PG)
*Ccajan*_61890_c0_g1_i1	−4.87	AT2G44480.5	5.12E-38	130	BGLU17	endo-1,3-beta glucosidase 17
*Ccajan*_7875_c0_g1_i1	−2.20	AT2G27500.1	0	524	BGLU14	endo-1,3-beta-glucosidase 14
*Ccajan*_2910_c0_g1_i1	−4.55	AT1G05990.1	7.56E-47	157	CML7	calmodulin-like protein 7
*Ccajan*_47280_c0_g1_i1	−7.53	AT4G00330.2	1.71E-93	285	CRCK2	calmodulin-binding receptor-like protein 2
*Ccajan*_41720_c0_g1_i1	−6.59	AT2G38110.1	1.18E-05	41.6	GPAT6	glycerol-3-phosphate acyltransferase 6 (GPAT6)
*Ccajan*_84874_c0_g1_i1	−6.27	AT1G06520.1	1.15E-11	58.5	GPAT1	glycerol-3-phosphate acyltransferase 1 (GPAT1)
*Ccajan*_13394_c0_g1_i1	−8.53	AT2G27110.3	1.48E-20	91.7	FAR3	fatty acid reductase 3 (FAR3)
*Ccajan*_697_c0_g1_i1	−2.33	AT1G61370.2	3.66E-11	57		S-locus lectin protein kinase family protein
*Ccajan*_52697_c0_g1_i1	−4.57	AT1G32560.1	3.64E-11	60.1	LEA6	Late embryogenesis abundant protein 6
*Ccajan*_51888_c0_g1_i1	−4.61	AT2G20635.1	2.68E-154	447	MAD1	mitotic spindle assembly checkpoint protein (MAD1)
*Ccajan*_41460_c0_g1_i1	−5.86	AT4G33270.1	4.07E-73	228	CDC20–1	Cell division cycle protein 20.1
*Ccajan*_41720_c0_g1_i1	−6.60	AT2G38110.1	1.18E-05	41.6	GPAT6	Glycerol-3-phosphate 2-O-acyltransferase 6
*Ccajan*_47215_c0_g1_i1	−7.14	AT2G43140.1	1.30E-20	90.5		Basic helix-loop-helix (bHLH) DNA-binding protein
*Ccajan*_68741_c0_g2_i1	−2.09	AT1G10610.1	6.30E-16	70.5	BHLH90	Transcription factor bHLH90
*Ccajan*_45806_c0_g1_i1	−3.53	AT3G19740.1	0	1237		P-loop containing nucleoside triphosphate hydrolases
*Ccajan*_697_c0_g1_i1	−2.33	AT1G61370.2	3.66E-11	57		S-locus lectin protein kinase family protein
**Up-regulated**						
*Ccajan*_38216_c3_g1_i1	7.25	AT1G54860.1	1.32E-35	128		GPI-anchored-like proteins
*Ccajan*_1528_c0_g3_i1	8.3	AT1G13710.1	9.73E-132	387	CYP78A5	Cytochrome P450-like 78A5
*Ccajan*_88538_c0_g1_i1	8.23	AT3G26300.1	3.88E-54	181	CYP71B34	Cytochrome P450-like 71B34
*Ccajan*_16351_c0_g1_i1	8.81	AT5G05690.3	2.13E-06	45.4	CYP90A1	cytochrome P450 90A1-like
*Ccajan*_55694_c0_g9_i1	6.36	AT1G50560.1	1.1E-18	80.5	CYP705A25	cytochrome P450 705A25-like
*Ccajan*_24040_c0_g3_i1	10.42	AT1G05840.1	2.7E-161	471		aspartic protease
*Ccajan*_37598_c0_g2_i1	9	AT5G47230.1	7.34E-42	148	ERF5	ethylene-responsive transcription factor 5 (*ERF5*)
*Ccajan*_37057_c0_g1_i1	8.99	AT4G24470.3	8.66E-75	241	ZIM	GATA transcription factor 25-like
*Ccajan*_35517_c1_g2_i5	2.55	AT5G49880.1	0	757	MAD1	mitotic spindle assembly checkpoint protein (MAD1)
*Ccajan*_36686_c0_g1_i4	10.05	AT2G38470.1	2.07E-39	142	WRKY33	WRKY transcription factor 33 (*WRKY33*)

^a^QueryId for homologue search using Blast

^b^Log2FC ≥ 2 represented up-regulated and Log_2_FC ≤ −2 were represented down-regulated

^c^*Arabidopsis* male-sterility/male-reproduction related genes in TAIR database (http://www.arabidopsis.org)

Transcription factors (TFs) are of major importance as they regulate gene expression in plants. Alterations in the gene expression are associated with modifications in the expression of transcription factors [67]. In our study, there were 16 DEGs representing transcription factors, 9 DEGs were down-regulated and 7 were up-regulated in the AKCMS11. The 9 down-regulated DEGs included 2 bHLH family, 2 C_2_H_2_zinc-finger, 2 basic-leucine zipper transcription factor (bZIP), 1 dof zinc-finger and 2 NAC domain-containing transcription factors. Among the 7 up-regulated DEGs, 1 were bHLH, 2 MYB transcription factors, 2 basic-leucine zipper transcription factors (bZIP), 1 NAC domain-containing transcription factorsand 1 WRKY transcription factor. (Additional file 2: Table S5).

### Metabolic pathways

Carbon fixation and energy metabolism are the most predominant metabolic pathways which are primarily responsible for furnishing the requirement of energy and carbon sources in the plants [68]. In the present study, 12 DEGs were observed to be participating in carbohydrate metabolism, 6 DEGs related to glycolysis/gluconeogenesis pathway and 6 DEGs related to pentose and glucuronate interconversions, respectively (Table 4). With respect to glycolysis, 4 DEGs were down-regulated and 2 DEGs were up-regulated in AKCMS11 in comparison to AKPR303. Additionally, among the 6 DEGs representing pentose and glucuronate interconversions, 3 DEGS were down-regulated and 3 DEGs were up-regulated in AKCMS11 (Table 4).

**Table 4:** KEGG pathways enriched of differentially expressed genes (DEGs) between sterile AKCMS11 and fertile AKPR303

Query ID	Subject Id	Gene Annotation	^a^Log_2_FC	^b^Regulation	^c^*p*-value
**Glycolysis/Gluconeogenesis**					
Ccajan_36409_c0_g2_i1	AT4G37870.1	Phosphoenolpyruvate carboxykinase	−2.33	down	0.04335
Ccajan_34307_c0_g1_i1	AT1G08650.1	Phosphoenolpyruvate carboxykinase	−2.23	down	0.043091
Ccajan_37543_c0_g2_i4	AT3G30841.2	Phosphoglyceratemutase	−2	down	0.003866
Ccajan_34880_c0_g2_i1	AT1G43670.1	Fructose-1,6-bisphosphatase	2.57	up	0.0418388
Ccajan_39910_c0_g1_i1	AT1G23190.1	phosphoglucomutase	−7.33	down	0.00156436
Ccajan_88886_c0_g1_i1	AT4G00570.1	NAD-dependent malic enzyme 2	2.28	up	0.04124277
**Pentose and glucuronate interconversions**					
Ccajan_36483_c0_g1_i4	AT2G37770.2	aldo-keto reductase family 4	−8.54	down	0.00872853
Ccajan_32476_c1_g2_i1	AT5G62420.1	Aldo/keto reductase family protein	8.47	up	0.0292025
Ccajan_30463_c0_g1_i1	AT3G18660.3	UDP-glucuronosyltransferase 1	8.83	up	0.0111043
Ccajan_18517_c0_g1_i1	AT1G22360.1	UDP-glycosyltransferase 85A2	−3.28	down	0.00017461
Ccajan_35958_c0_g1_i1	AT3G16520.2	UDP-glycosyltransferase 88A1	−8.17	down	0.00053557
Ccajan_38121_c2_g1_i5	AT3G53150.1	UDP-glucosyl transferase 73D1	4.24	up	0.0002212
**Tricarboxylic acid cycle**					
Ccajan_64232_c0_g1_i1	AT1G79750.1	Malate dehydrogenase	−6.46	down	0.001118
Ccajan_35485_c0_g1_i1	AT2G20420.1	Succinate-CoA synthetase	2.30	up	0.00176114
**Starch and sucrose metabolism**					
Ccajan_7875_c0_g1_i1	AT2G27500.1	Glucan endo-1,3-beta-glucosidase 14	−2.20	down	0.01872407
Ccajan_61890_c0_g1_i1	AT2G44480.5	beta glucosidase 17	−4.87	down	0.02273399
**Alpha-linolenic acid metabolism**					
Ccajan_25458_c0_g1_i1	AT3G51840.1	Acyl-coenzyme A oxidase 4	3.47	up	0.02874461
**Oxidative phosphorylation**					
Ccajan_34619_c0_g1_i1	AT4G38920.1	Vacuolar H^+^-ATPase subunit C1	−9.93	down	1.84E-05
Ccajan_36113_c2_g1_i1	AT4G11150.1	Vacuolar H^+^-ATPase subunit E1	−10.69	down	0.001949
Ccajan_34619_c0_g3_i1	AT4G38920.1	Vacuolar H^+^-ATPase subunit C1	10.2	up	7.45E-05
Ccajan_36113_c2_g3_i2	AT4G11150.1	Vacuolar H^+^-ATPase subunit E	3.365	down	0.015683
Ccajan_58593_c0_g1_i1	AT5G21430.1	NADH dehydrogenase subunit B2	−6.28	down	0.001759
Ccajan_41295_c0_g1_i1	ATMG01360.1	Cytochrome c oxidase subunit 1	−6.46	down	0.00170655
Ccajan_13673_c0_g1_i1	AT1G22450.1	cytochrome c oxidase subunit 6b	−5.98	down	0.000415
**Reactive oxygen species (ROS) generation/scavenging**					
Ccajan_35344_c0_g3_i4	AT5G06720.1	peroxidase 53	−3.05	down	0.005337
Ccajan_12688_c0_g1_i1	AT5G05340.1	peroxidase 52	−8.62	down	8.82E-05
Ccajan_20878_c0_g1_i1	AT1G65980.1	peroxiredoxin-2B-like	−4.41	down	0.045412
Ccajan_68963_c0_g2_i1	AT3G10920.2	superoxide dismutase	−11.65	down	1.44E-11
Ccajan_34224_c0_g1_i2	AT5G21105.1	ascorbate oxidase	8.35	up	0.03753425
Ccajan_90965_c0_g1_i1	AT4G31870.1	glutathione peroxidase 7	−5.20	down	0.02719158
Ccajan_36196_c0_g2_i3	AT2G30870.1	glutathione s-transferase F10	2.41	up	0.02883
Ccajan_12449_c0_g1_i1	AT5G18600.1	Monothiol glutaredoxin-S2	−2.23	down	0.02449176
Ccajan_18941_c0_g2_i1	AT4G15680.1	Monothiol glutaredoxin-S4 (GRXS4)	−8.66	down	0.00724279
Ccajan_20878_c0_g1_i1	AT1G65980.1	Peroxiredoxin-2B	−4.41	down	0.04541246
Ccajan_37604_c0_g1_i5	AT3G62930.1	Monothiol glutaredoxin-S6 (GRXS6)	−4.45	down	0.00130434
Ccajan_82144_c0_g1_i1	AT5G39950.1	Thioredoxin H2	−6.43	down	0.00129587
Ccajan_18941_c0_g3_i1	AT4G15680.1	Monothiol glutaredoxin-S4 (GRXS4)	9.04	up	0.00570316
Ccajan_24088_c0_g1_i1	AT4G33040.1	Glutaredoxin-C6	4.08	up	0.04803176
Ccajan_37604_c0_g1_i3	AT3G62930.1	Monothiol glutaredoxin-S6 (GRXS6)	10.06	up	0.01361605

^a^Log_2_FC ≥ 2 represented up-regulated and Log_2_FC ≤ −2 were represented down-regulated

^b^Regulation direction of the DEGs, AKPR303 restorer was the control

^c^*p*-value of ≤0.05 was considered statistically significant

In this study, 2 DEGs participated in the Tricarboxylic acid cycle (TCA), 1 DEG encoding malate dehydrogenase and second encoding succinate-CoA synthetase (Table 4). Also, we detected 7 DEGs involved in oxidative phosphorylation, majority of the DEGs showed down-regulation with only 2 DEGs showing up-regulation in the AKCMS11 (Table 4). We examined a total of 15 DEGs involved in the elimination of reactive oxygen species (ROS), including 2 DEGs encoding peroxidase-like, 1 DEG encoding superoxide dismutase and 2 DEG encoding peroxiredoxin-2B-like, along with 1 DEG encoding glutathione S-transferase and 1 DEG encoding for glutathione peroxidase. Additionally, there were 5 DEGs encoding for monothiol glutaredoxin, and 1 DEG each encoding for ascorbate oxidase, thioredoxin and glutaredoxin. 10 of the DEGs showed down-regulation in AKCMS11 in comparison to AKPR303 (Table 4).

### Confirmation of DEGs by qRT-PCR

**Fig. 11:**
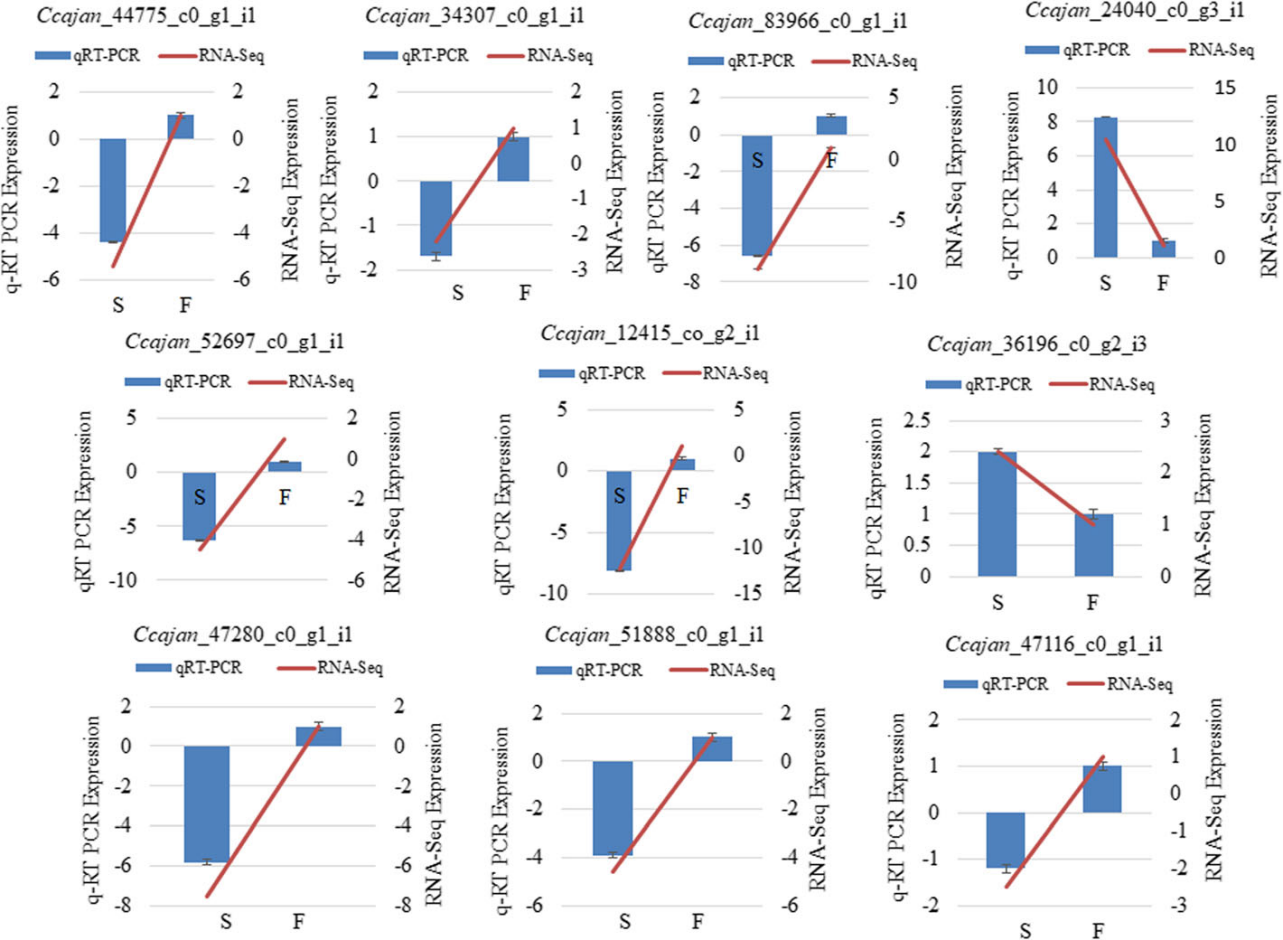
qRT-PCR validation of differentially expressed unigenes. S denotes the sterile AKCMS11 and F denotes the fertile AKPR303. The primary vertical axis represents qRT-PCR expression values and secondary vertical axis represents RNA-Seq expression values

**Fig. 12:**
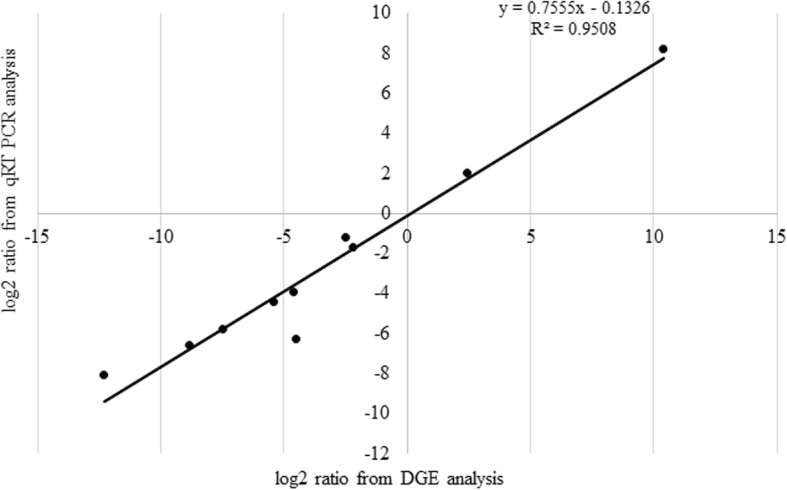
Linear regression analysis of the fold change of the gene expression ratios between DEG data and qRT-PCR. 10 primers were selected for qRT-PCR analysis to confirm the accuracy and reproducibility of the Illumina expression. Scatterplots were generated by the log_2_ expression ratios from DGE sequencing data (x- axis) and qRT-PCR data (y-axis)

We randomly selected 10 genes for qRT-PCR analysis with the aim to validate the expression profiles between AKCMS11 and AKPR303 obtained by RNA-Seq. The list of DEGs specific primers used for qRT-PCR analysis is listed in Additional file 2: Table S12. The DEGs selected for qRT-PCR confirmation were related to callose synthase 7-like, phosphoenolpyruvate carboxykinase, polygalacturonase, aspartic protease, late embryogenesis abundant (LEA), uncharacterized mitochondrial protein, glutathione S-transferase F10, calmodulin binding receptor-like protein, mitotic spindle assembly checkpoint protein (MAD1), and pentatricopeptide repeat-containing protein. Although the relative gene expression detected by RNA-Seq analysis was higher than those detected by qRT-PCR, the overall expression patterns were similar (Fig. 11 and Table 5). Linear regression analysis was performed and it revealed positive correlation (R^2^ = 0.9508) between the qRT-PCR and DGE fold change gene expression ratios, indicating that the expression of all the 10 genes depicted by qRT-PCR analysis was in agreement with the DGE data analysis (Fig. 12). These findings confirm the reliability of the Illumina RNA-Seq data and its analysis.

**Table 5:** Confirmation of the expression profiles of selected genes by qRT-PCR

Gene Id	Description	Fold change	
		RNA-Seq	qRT-PCR
*Ccajan*_44775_c0_g1_i1	callose synthase 7-like	−5.4	−4.4
*Ccajan*_34307_c0_g1_i1	phosphoenolpyruvate carboxykinase	−2.2	−1.7
*Ccajan*_83966_c0_g1_i1	Polygalacturonase (*PG*)	−8.9	−6.6
*Ccajan*_24040_c0_g3_i1	aspartic protease	10.4	8.2
*Ccajan*_52697_c0_g1_i1	Late embryogenesis abundant protein 6 (*LEA6*)	−4.5	−6.3
*Ccajan*_12415_c0_g2_i1	uncharacterized mitochondrial protein	−12.3	−8.1
*Ccajan*_36196_c0_g2_i3	glutathione s-transferase F10	2.41	2.0
*Ccajan*_47280_c0_g1_i1	calmodulin binding receptor-like protein	−7.5	−5.8
*Ccajan*_51888_c0_g1_i1	mitotic spindle assembly checkpoint protein (*MAD1*)	−4.6	−3.9
*Ccajan*_47116_c0_g1_i1	pentatricopeptide repeat-containing protein	−2.5	−1.2

Fold change refers to expression in sterile AKCMS11 in comparison to restorer AKPR303; Negative value refers down-regulated expression in sterile AKCMS11

## Discussion

**Fig. 13:**
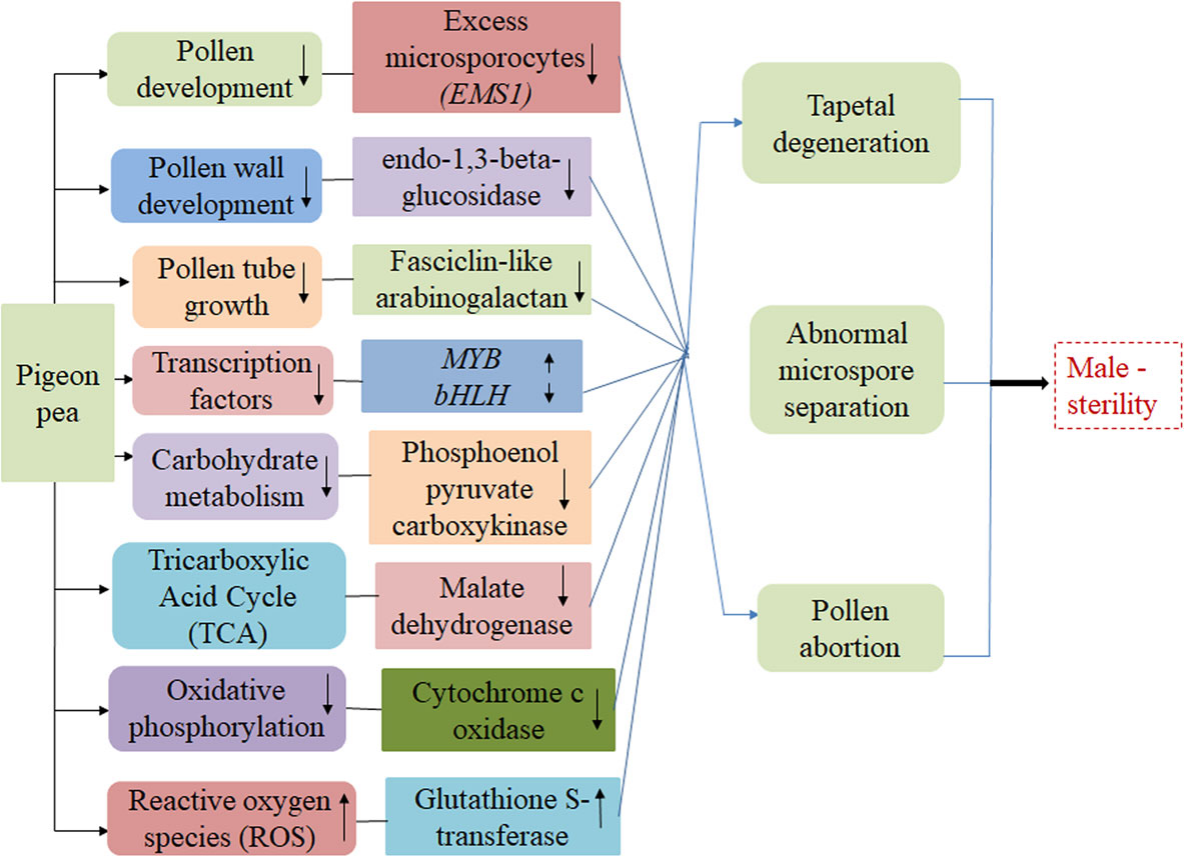
Some of the candidate differentially expressed genes (DEGs) involved in cytoplasmic male-sterility sterility in pigeon pea. The upper arrows indicate up-regulation of the DEGs, and the down arrows represent down-regulation of the DEGs. Each color box depicts an individual metabolism

Cytoplasmic male-sterility (CMS) is considered as an important tool for hybrid seeds production in many plant species. To elucidate the underlying mechanism governing CMS in pigeon pea, the present study was undertaken. Comparative transcriptome sequencing and analysis of the floral buds from sterile (AKCMS11) and fertile (AKPR303) pigeon pea were performed. In total, 3167 genes showed differential expression between the CMS and the fertile restorer. Based on the functional annotation, we identified some candidate DEGs related to pollen development and metabolism which were previously reported to be involved in male-sterility (Fig. 13).

### DEGs involved in pollen development potentially related to CMS

We identified 34 pollen development related genes which were found to have homologs in *Arabidopsis* (Table 3). Pollen formation is a critical developmental mechanism in plants which is highly dependent on co-ordinated metabolic pathways [69]. Male-sterility occurs due to the aberrant functioning of the genes responsible for tapetum development, pollen formation, callose degradation and pollen wall development [70]. In our findings, several DEGs with a potential role in pollen development were observed (Table 3). Pollen development is an intricate and programmed process which takes place within specialized structures called anthers from reproductive cells (microsporocytes). These microspores (pollens) are dependent on the sporophytic tissues for their development, finally leading to the release of functional pollens for fertilization in flowering plants [71]. In *Arabidopsis*, *EXCESS MICROSPOROCYTES 1 (EMS1)* proposes a leucine-rich repeat receptor protein kinase (LRR-RPK) which is actively involved in the determination and differentiation of the tapetal cells and the microsporocytes during plant reproduction. It was observed that the anthers of the *EMS* mutant (*EMS1*) exhibited excessive microsporocytes despite lacking the tapetum, producing non-functional pollens and leading to male-sterility [22]. We also observed 1 DEG (*Ccajan*_10186_c0_g1_i1) encoding leucine-rich repeat receptor protein kinase *EMS1*, down-regulated (4.53-fold) in the AKCMS11 respectively and the gene thus may also be related to pollen development and fertility in pigeon pea.

During the process of pollen development, tapetum is of great significance as it provides nourishment to the developing microspores leading to pollen wall formation [72]. In *Arabidopsis*, *MALE STERILITY1* (*MS1*) and *MALE SERILITY2* (*MS2*) genes regulate the stages of tapetum maturation and pollen wall biosynthesis and ending with the release of viable pollen [27, 73]. The *MS1* gene exhibits homology to PHD-finger transcription factor critical for tapetum and pollen development [74]. A PHD-finger transcription factor *MALE STERTILITY1* (*MS1*) conferring male-sterility was identified in barley [75]. In this study, 1 DEG (*Ccajan*_34303_c0_g2_i2) encoding PHD-finger transcription factor *MALE STERTILITY1* (*MS1*) was detected with 4.27-fold down-regulation in the AKCMS11. In the *MS1* mutant, immediately after the release of microspores from the tetrads, sudden preterm degeneration of the tapetum and pollen takes place leading to inviable pollen formation and cause male-sterility [27]. Similar results were reported in *male-sterile* tomato mutants *ms3*, *ms15*, *ms5* and *ms1035* due to dysfunctional regulation of the tapetum [57, 76, 77].

In flowering plants, pollens are secured within the pollen grains and physically protected by the complex pollen cell walls. The pollen mother cells (PMC) synthesize a polysaccharide, callose (β-1,3-glucan) and the outer protective callose wall segregates the newly developed microspore and restricts them from merging with the neighbouring tapetum tissues, giving the unique tetrad shape [78]. Finally, the callose wall surrounding the microspores degenerates, releasing them into the anther locule [79]. The callose synthase enzyme is required for the synthesis of a temporary callose wall around the developing microspore, twelve *callose synthase* (cals*) genes, are reported in Arabidopsis* [80, 81]. The callose synthase 5 (cals5) mutants showed the absence of callose deposition around the microspores, confirming the importance of this enzyme with reduced fertility [38]. Interestingly, we noticed 2 DEGs; *Ccajan*_44775c0_g1_i1 and *Ccajan*_52234_c0_g1_i1 representing callose synthase with 5.45 and 6.38-fold down-regulation in the AKCMS11 in comparison to the AKPR303 thus featuring as a prominent candidate in pollen development. These results are consistent with the earlier reports in watermelon and soybean [55, 56]. Additionally, primexine a microfibrillar polysaccharide matrix, is important for the normal development of functional pollen and is regulated by an *AUXIN-RESPONSIVE FACTOR* (*ARF17*). In *Arabidopsis*, *ARF17* mutant displayed abnormal callose wall formation with no primexine and pollen abortion [82]. A single DEG *Ccajan*_41968_c0_g1_i1 for *ARF72* displayed reduced expression of 6.21-fold in the AKCMS11 thus featuring as a prominent candidate in pollen development.

Sporopollenin is the key constituent of the outer rigid exine wall of the microspores and genes like *CYTOCHROME P450* (*CYP703A2*), *CYTOCHROME P450* (*CYP704B1*) and acyl-coA synthetase 5 (acos5) are responsible for regulating the biosynthesis of sporopollenin. Down-regulation of the *CYP703A2* and *CYP704B1* genes depicted the reduced level of sporopollenin, an absence of outer exine layer with unique stripped surface and impaired pollens in *Arabidopsis* [34, 35, 73]. Surprisingly, 4 genes corresponding to *CYTOCHROME P450-like* were drastically over-expressed in the AKCMS11 in comparison to AKPR303. For example, *Ccajan*_1528_c0_g3_i1 (8.30-fold), *Ccajan*_88538_c0_g1_i1 (8.23-fold), *Ccajan*_16351_c0_g1_i1 (8.81-fold) and *Ccajan*_55694_c0_g9_i1 (6.36-fold). This finding is supported by a previous study in cotton where they proposed that since these genes are involved in the synthesis of sporopollenin, their up-regulation must have ended up in immoderate accumulation sporopollenin and ultimately male-sterility [83]. Over-expression of this gene may be related to pollen abortion.

The developing microspores (pollen grains) within the anthers are enclosed by a layer of cells known as tapetum [84, 85], it is of considerable importance as it provides the necessary nourishment to the developing pollens and secretes components for the outer exine and regulates the pollen wall formation [86, 87]. During the subsequent later stages of pollen development, tapetum experiences a regulated disintegration by programmed cell death (PCD) and releases all the cellular components required for pollen wall formation into the anther locule [88–90]. Any delay in the timely regulation of tapetum PCD results in pollen lethality and male-sterility [87]. Previously, a *PCS1* gene encoding an anti-cell-death factor known as an aspartic protease which governs PCD in *Arabidopsis* was reported [91]. The over-expression of the *PCS1* gene produced excessive aspartic protease which further inhibited anther dehiscence and resulted in male-sterility. In this study, 1 DEG (*Ccajan*_24040_c0_g3_i1) was 10.42-fold over-expressed in the sterile AKCMS11 than the fertile AKPR303. The higher expression in the sterile line could have delayed the PCD of the tapetum, which is a must for anther dehiscence and ultimately lead to pollen sterility in pigeon pea. A similar observation was seen in soybean which therefore stands in support of the present finding [56].

In this study, 3 DEGs related to cell wall modification were identified and all were strongly down-regulated in the AKCMS11 as compared to the AKPR303. These genes encode cell wall-degrading enzymes such as polygalacturonase (PG), known for hydrolyzing pectin and polygalactouronic acid and endo-1,3-beta-glucosidase-like, cellulose- hydrolyzing enzyme. In an earlier report in *Arabidopsis*, *QRT1* and *QRT2* mutant produced non-functional pollen due to the inefficiency of polygalacturonase in pectin degradation around the microspores [92]. A single DEG (*Ccajan*_83966_c0_g1_i1) representing polygalacturonase (PG) showed 8.85-fold down-regulation with 2 DEGs (*Ccajan*_61890__c0_g1_i1 and *Ccajan*_7875_c0_g1_i1) encoding endo-1,3-beta-glucosidase-like were 4.87-fold and 2.20-fold down-regulated in the AKCMS11, respectively. The results were consistent with the findings in tomato and cotton [11, 93]. These under-expressed genes might be involved in pollen abortion.

Arabinogalactan glycoproteins (AGPs) are present in different cells and tissues of the higher plants and are actively involved in the growth and reproduction [94]. In *Arabidopsis*, AGP6, AGP11 and Fasciclin-like arabinogalactan proteins (FLA3) are the key genes involved in the synthesis of AGPs [95, 96] and are in primarily expressed in the pollen grains and pollen tube with their participation in the microspore development. Alterations in the genes result in obstruction in pollen tube growth and defective pollen release from the anthers leading to reduced fertility [95, 96]. In our results, we identified 3 differentially expressed transcripts encoding arabinogalactan proteins (AGPs) such as GPI-anchored like protein and Fasciclin-like arabinogalactan 11 (FLA11). Of the 2 DEGs encoding GPI-anchored like proteins, 1 was 5.0-fold down-regulated (*Ccajan*_38216_c1_g2_i3) in CMS genotype while the other gene showed up-regulation (7.25-fold). The DEG *Ccajan_*37653_c0_g4_i5 represents FLA11 with 9.14-fold down-regulation in CMS line with respect to the sterile line. Similar results were observed in watermelon, cotton and sesame [55, 61, 97].

Calcium ions (Ca^2+^) ions are known for their considerable physiological significance in plants [98]. They are actively involved in plant reproduction, specifically participating in pollen germination and pollen tube growth. It was earlier reported that sufficient Ca^2+^ ions result in normal pollen germination while any changes in the concentration (higher or lower) restrain pollen germination and tube elongation [99, 100]. In the present study, 2 DEGs *Ccajan*_47280_c0_g1_i1 and *Ccajan*_2910_c0_g1_i1 representing calmodulin-binding receptor-like proteins were identified, 1 showing 4.55-fold down-regulation and the other 7.53-fold down-regulation. Altered expression of these genes in the sterile AKCMS11 might have inhibited pollen germination and tube growth. Similar reports in watermelon, cotton and kenaf support the present results [55, 93, 101].

During the final stages of pollen maturation, the pollen dehydrates, and the mature pollen is ready for germination [102]. Late embryogenesis abundant (LEA) proteins are involved in conferring desiccation tolerance to the pollen. In lily, *LP28* is a pollen-specific LEA-like protein is known which slowly accumulates in the developing pollen and generously present in the germinated pollen after hydration with their probable role in pollen maturation and pollen tube growth [103]. In this investigation, only 1 DEG was found related to *LEA*-like protein, for example, *Ccajan*_52697_c0_g1_i1 showing 4.57-fold down-regulation in the CMS line. Abnormal expression of this gene may be related to pollen development. The results are in accordance with the results in watermelon and soybean [55, 56].

Male gametogenesis is a complex process in flowering plants with rounds of mitotic and meiotic cell divisions leading to the development of male gametophyte. Any abnormality in the genes regulating the cell division can cause male-sterility [104]. In this study, 2 mitotic spindle assembly checkpoint protein (MAD1) genes and 1 cell division protein gene displayed abnormal expression in the sterile AKCMS11 in comparison to fertile restorer AKPR303. A similar finding was reported in watermelon [55] suggesting it might be involved in pollen development. The findings strongly indicate the potential role of these genes responsible for pollen development leading to CMS in pigeon pea.

### DEGs encoding transcription factors potentially related to CMS

Transcription factors (TF’s) are proteins which bind to *cis-*regulatory specific sequences in the promoter region of the target genes and regulate the gene expression [105]. Any alterations in the expression of transcription factors lead to change in the gene expression causing substantial transformation during plant development [106]. In *Arabidopsis***,** 608 transcription factors (TFs) belonging to 34 families have been described to be participating in pollen development [107]. In our study, 9 and 7 DEGs encoding transcription factors showed down-regulation and up-regulation in the sterile AKCMS11 in comparison to its fertile restorer, respectively. MYB proteins constitute a large and functionally diverse family with a major role in plant-specific processes [108]. In *Arabidopsis*, *AtMYB32* and *AtMYB103* are expressed in the tapetum and facilitate the development of pollen by controlling tapetum development, callose dissolution and exine formation [28, 29]. Any changes in their expression can lead to early tapetal degeneration, distorted pollens and male-sterility [28, 109]. In this study, 2 DEGs *Ccajan_*30780_c0_g1_i2 and *Ccajan*_36059_c0_g1_i1 encoding *MYB* transcription factors were identified, both up-regulated (3.59-fold and 3.11-fold) in the AKCMS11. Thus pointing towards the possible role of these differentially expressed genes in pollen development.

Transcription factors like bHLH are significant as they synchronize the process of normal tapetum development in the anthers. bHLH- type transcription factors such as *DYT1* and *AMS* in *Arabidopsis* [110] and *UNDEVELOPED TAPETUM* (*OsUDT1*) in rice [111] are known to be crucial for proper tapetum development and finally leading pollen development. In our data, we observed 3 DEGs encoding bHLH transcription factors among those 2 DEGs showed down-regulation in the CMS line. For instance, *Ccajan*_47215_c0_g1_i1 was 7.14-fold down-regulated and *Ccajan*_68741_c0_g2_i1 was 2.08-fold down-regulated. Similar findings were seen in radish, sesame and kenaf [54, 97, 101].

In *Arabidopsis*, WRKY transcription factors like *WRKY2*, *WRKY34* and *WRKY 27* are known for their candidate role in pollen development during male gametogenesis [112]. Over-expression of *WRKY 27* showed limited pollen viability leading to male-sterility [113]. In our study, there was 1 DEG which was over-represented in the AKCMS11; *Ccajan*_36686_c0_g1_i4 (10.05-fold up-regulated), this was in accordance with findings in soybean and sesame [56, 97]. These DEGS recognized in this study might have a possible role causing CMS in this crop.

### DEGs involved in carbohydrate metabolism potentially related to CMS

In plants, carbohydrates in the form of sugars and starch are the substrate for energy supply and growth. Sufficient sugar level is highly important for the anther development and subsequently during pollen maturation sugars get converted into starch which later serves as the energy source for pollen germination [114, 115]. Dysfunctional sugar metabolism can significantly influence pollen development producing impaired pollens and results in male-sterility [116]. This condition was observed in pepper and cabbage [117, 118]. In this study, altered expression of genes involved in glycolysis, gluconeogenesis and pentose and glucuronate interconversions were detected in the AKCMS11 in comparison to the AKPR303 (Table 4). Total 6 DEGs were involved in glycolysis and gluconeogenesis, for instance *Ccajan_*36409_c0_g2_i1, *Ccajan*_34307_c0_g1_i1, *Ccajan*_37543_c0_g2_i4, *Ccajan*_34880_c0_g2_i1, *Ccajan*_39910_c0_g1_i1 and *Ccajan*_88886_c0_g1_i1, all of them were significantly down-regulated in the sterile AKCMS11 except for 1 DEG which showed up-regulation (Table 4). 6 DEGs were related to pentose and glucuronate interconversions, 3 of which were down-regulated and 3 DEGs were up-regulated in AKCMS11. These genes involved in carbohydrate metabolism exhibited lower expression suggesting the low availability of sugars during pollen development resulting in non-functional pollens and male-sterility. The results were in accordance with the previous report in soybean, chili pepper and cotton [56, 59, 62].

### DEGs involved in the tricarboxylic acid cycle (TCA) and oxidative phosphorylation potentially related to CMS

Mitochondria are the main energy yielding-power houses of the cells with a number of important metabolic pathways, involving TCA cycle, respiratory electron transfer, and oxidative phosphorylation [119–121]. Pollen formation is an energy-utilizing process which is highly dependent on mitochondrial respiration and fermentation for satisfying their energy demands [102, 122]. We identified, 2 DEGs participating in the TCA cycle, *Ccajan*_64232_c0_g1_i1 encoding malate dehydrogenase and *Ccajan*_35485_c0_g1_i1 encoding succinate-CoA synthetase (Table 4). According to the previous reports in cotton, *Brassica napus* and cabbage lower expression of TCA related genes causes pollen abortion and are responsible for CMS [93, 123, 124]. These observations suggest that alterations in the energy-yielding metabolic pathways inhibits pollen development and may be the cause of CMS.

The mitochondria generate energy in the form of adenosine triphosphate (ATP) by utilizing two major pathways, the respiratory electron transfer chain and oxidative phosphorylation [125]. In this study, 7 genes related to respiratory electron transfer chain and oxidative phosphorylation were detected showing differential expression in the sterile AKCMS11 in comparison to the fertile AKPR303 (Table 4). Cytochrome c oxidase (cox) is the key enzyme involved in the last stage of the mitochondrial electron transport chain and considered as a major regulating site during ATP synthesis [126]. Previous literature on rice and cotton proposed the importance of cox genes in conferring CMS [127, 128]. Two DEGs *Ccajan*_13673_c0_g1_i1 and *Ccajan*_41295_c0_g1_i1 encoding cytochrome c oxidase subunit 6b and subunit 1 were 5.98-fold and 6.46-fold down-regulated in the AKCMS11 (Table 4). This was in accordance with the earlier reports in chili pepper, cotton, *Brassica napus* and beet [59, 93, 123, 129]. Hence, altered expression of cox 6 might have resulted in decreased ATP synthesis and disrupted the pollen formation in this crop.

### DEGs involved in the elimination of reactive oxygen species (ROS) potentially related to CMS

In plants cells, reactive oxygen species (ROS) are the by-products of aerobic respiration, constantly being generated in the chloroplast, mitochondria, and peroxisomes and are maintained by ROS scavenging system [130]. The ROS are free radicals including superoxide (O^2−^), hydrogen peroxide (H_2_O_2_), and malondialdehyde (MDA) and their excessive accumulation in the cells can instigate cell apoptosis [131]. Previously, excessive accumulation of the ROS species and remarkable reduction of the antioxidative defense systems in the anthers of the cotton CMS line caused male-sterility [132]. In rice, the abnormal concentration of the ROS in the mitochondria induced acute oxidative stress during pollen development and leading to male-sterility [133]. In this study, 15 DEGs involved in the elimination of ROS were detected with 5 DEGs showing over-expression and 10 DEGs down-expression in the sterile AKCMS11 line in comparison to the fertile AKPR303 (Table 4). Down-regulation of the genes encoding oxygen scavenging enzymes in AKCMS11 triggered the excessive accumulation of ROS in the sterile buds, leading to pollen abortion [134]. In broccoli, higher-expression of glutathione S-transferase (GST) gene induced excessive ROS and resulted in male-sterility [70]. Interestingly, we came across 1 DEG encoding glutathione S-transferase (GST) for instance, *Ccajan*_36196_c0_g2_i3, showing significant higher-expression 2.41-fold in AKCMS11 in comparison to AKPR303. We presume, expression alterations of the genes involved in ROS scavenging probably induced pollen abortion in pigeon pea.

## Conclusions

To our knowledge, this is the first reported attempt at transcriptome sequencing and comparative analysis of the floral buds of A_2_ CMS system (*Cajanus scarabaeoides* L.) derived cytoplasmic male-sterile (AKCMS11) and its fertility restorer (AKPR303) in pigeon pea. The differential gene expression analysis showed 3167 differentially expressed genes (DEGs) between AKCMS11 and AKPR303, among which 1432 were up-regulated and 1390 were down-regulated in the AKCMS11 when compared to the AKPR303. Based on the functional annotation of these DEGs in GO, KEGG databases and metabolic pathway analysis combined with previous studies, we hereby conclude that male-sterility of AKCMS11 is probably related to abnormalities of some of the putative DEGs in functional and metabolic pathways, such as involved in pollen development, encoding transcription factors, elimination of reactive oxygen species (ROS), carbon metabolism, oxidative phosphorylation, tricarboxylic acid cycle (TCA), etc. Further studies of these crucial genes will focus on clearly understanding their functions in conferring male-sterility. Therefore, this present report will be highly informative and provide support for future studies in elucidating the molecular basis related to cytoplasmic male-sterility in pigeon pea.

## Methods

### Plant material and RNA isolation

Cytoplasmic male-sterile AKCMS11 (*Cajanus scarabaeoides* cytoplasm) and fertility restorer line AKPR303 (*Cajanus cajan* cytoplasm) [47] of pigeon pea were used in this present investigation as described earlier [135]. The seeds for both the lines were procured from Pulse Research Unit, Dr. Panjabrao Deshmukh Krishi Vidyapeeth, Krishi Nagar, Akola, Maharashtra, India. The seeds were sown directly in the soil and the plants were maintained under natural conditions in the experimental greenhouse at ICAR- NIPB, New Delhi. Flower buds of different-sized were collected from two independent plants (representing replications) of each AKCMS11 and AKPR303 lines, respectively. The young buds were harvested, frozen in liquid nitrogen and stored at − 80 °C. Total RNA was extracted from the flower buds of both the lines (in replicates) using Spectrum Plant Total RNA Kit (Sigma-Aldrich, USA) following the manufacturer’s protocol.

### Pollen fertility test

Pollen analysis was performed to evaluate pollen fertility in the male-sterile (AKCMS11) and fertile (AKPR303) buds. Anthers from the flower buds of AKCMS11 and AKPR303 were removed and squashed on a glass slide in 1% acetocarmine dye. Then the glass slide was examined under a microscope.

### Library preparation and RNA sequencing

Total RNA was checked on 1% denaturing agarose gel and quantified using Nanodrop spectrophotometer (Thermo Fischer Scientific, USA). The RNA quality was analyzed with RNA 6000 NanoAssay kit (Agilent Technologies, USA) using Agilent 2100 Bioanalyzer (Agilent Technologies, USA). Total four RNA libraries were constructed from the buds of sterile and fertile lines (two replicates each) using Truseq RNA Sample prep kit (Illumina, USA.) following the manufacturer’s protocol. The libraries were then sequenced by Illumina paired-end sequencing technology on Illumina HiSeq 1000 sequencer.

### RNA-Seq data analysis and de novo transcriptome assembly

Sequencing raw data was received in the FASTQ format. The per base sequence quality of the raw reads from sterile (41,531,572; 68,068,793) and fertile (29,618,927; 42,957,061) lines respectively were determined by FastQC version 0.11.4 (http://www.bioinformatics.babraham.ac.uk/projects/) [136]. Rcorrector software was also employed for checking the *k*-mer content in the data [137]. Raw reads with PHRED quality score < 30 and shorter than 50 bp in length were filtered and not considered for further transcriptome assembly. Trimmomatic software version 0.36 [138] was used for trimming of the raw reads to remove the adaptor sequence followed by filtering of low-quality reads (quality score ≤ 5) and reads with the unknown ‘N’ base (‘N’ ratio ≥ 10%) to finally obtain the filtered clean reads. Next, following the read orientation (R1 and R2) the total clean reads from the sterile and fertile lines were concatenated together by using an in-house shell script. A de novo assembly of the pooled clean reads was achieved by Trinity software version v2.2.0 [139] using default parameters. Further, Bowtie2 aligner was used for the validation of the transcriptome assembly by mapping back the filtered reads onto the assembled transcriptome. Then, we evaluated the completeness of our final transcriptome assembly with Benchmarking Universal Single-Copy Orthologs tool version 3 (BUSCO) [140, 141]. BUSCO is ideal for assessing assembly completeness, because the expectation that these genes are found in a given genome as single copies is reasonable from an evolutionary point of view [140–143]. For the present analysis, we used the transcriptome assessment mode with the eukaryote lineage database (eukaryota_orthoDB9) and viridiplantae lineage database (viridiplantae_orthoDB10). Subsequently, non-redundant unigenes were obtained by employing the CD-HIT software version 4.6.1with identity parameter of 95% (http://weizhongli-lab.org/cd-hit/) [144]. Followed by removal of rRNAs and other housekeeping non-coding RNAs (tRNAs, snRNAs, snoRNAs etc.) by BLASTN search against Rfam database [145]. GC content of the total unigenes was calculated via GC-Profile tool ^(^http://tubic.tju.edu.cn/GC-Profile/) [146].

### Functional annotation of the unigenes

Functional annotation of the assembled unigenes was achieved by BLASTX search against Nr (NCBI non-redundant protein sequences), Virdiplantae, GO (Gene ontology) and KEGG (Kyoto encyclopedia of genes and genomes) databases with a stringent cut-off E-value of 1e^− 3^ [147–149]. Unigenes annotation against Nr database was performed with the aid of Blast2GO tool version 3.1.3 (http://www.blast2go.org) [150] up to 10 qualified blast hits for each unigene. Among the 10 blast hits the one with higher sequence similarity was accounted as a significant match for each unigene. Transcription factors were identified with the help of PlantTFcat online tool (http://plantgrn.noble.org/PlantTFcat) [73].

### Differential gene expression analysis

Firstly, RSEM software [74] was used for calculating the read count of each uniquely mapped transcript on to the assembled transcriptome (for each sample) in terms of FPKM (fragments per kilo base per million). Then, differentially expressed genes (DEGs) between the sterile and fertile genotypes (two replications for each) were detected by DESeq2 [61]). Strict criteria of *P*-value and FDR (False discovery rate) ≤ 0.05 and Log_2_ FC (Fold change ratio sterile vs fertile) ≥ 2 and ≤ − 2 (for up-regulated and down-regulated genes) were used for determining significant differentially expressed genes (DEGs).

### GO and KEGG enrichment analysis of the DEGs

Gene ontology (GO) annotation of all the differentially expressed genes (DEGs) was performed using Singular Enrichment Analysis Tool in AgriGO software version 2.0 (http://bioinfo.cau.edu.cn/agriGO/) with default parameters [151]. Based on GO enrichment results, the ones with *P*-value ≤0.05 were considered as significant GO terms. Then, the functional GO classification of all the DEGs was performed by WEGO software (wego.genomics.org.cn/) and the results were categorized into three independent hierarchies; biological processes, molecular functions and cellular components. KEGG (Kyoto encyclopedia of genes and genomes) pathway enrichment analysis of all the DEGs was conducted by KEGG Mapper (http://www.genome.jp/kegg/mapper.html) with default parameters. The DEGs were compared to the entire reference gene set by hypergeometric tests to identify the pathways enriched for DEGs and a *P*-value ≤ 0.05 indicated significant pathway enrichment. Additionally, MapMan version 3.3. 0 [65] was used for metabolic pathway analysis of the DEGs by searching against *Arabidopsis thaliana* TAIR database (http://www.arabidopsis.org).

### Validation of DEGs by quantitative real time-PCR (qRT-PCR)

Total RNA was isolated from the buds of sterile (AKCMS11) and restorer (AKPR303) line as mentioned earlier. First-strand cDNA was prepared from 2 μg of total RNA using RevertAid First strand cDNA synthesis kit (Thermo Fischer Scientific, USA) according to manufacturer’s instructions. The first strand cDNA was then further diluted ten times for quantitative real-time PCR (qRT-PCR) reaction. qRT-PCR was performed using KAPA SYBR FAST qPCR Master Mix (2X) (KAPA Biosystems, USA) on ABI PRISM 7500 Real-Time PCR System (Applied Biosystems, USA). qRT-PCR was performed in three independent technical replicates for each genotype. The reaction mixture (20 μl) contained 3 μl of cDNA, 10 μl of 2X SYBR green qPCR master mix, 0.20 μl of primers (forward and reverse) and final volume was adjusted with nuclease-free water. PCR conditions were 94 °C for 3 min followed by 40 cycles of 94 °C for 3 s, 60 °C for 15 s and 72 °C for 15 s. α-*tubulin* was used as the internal reference gene. The relative level of gene expression was calculated using the 2^(−ΔΔCt)^ algorithm, fertile restorer (AKPR303) was used as a calibrator.

## Supplementary information

**Additional file 1: Table S1.** Rcorrector ouput for *k*-mer content in the raw paired-end data. **Table S2.** Bowtie2 alignment statistics in sterile (AKCMS11) and fertile restorer (AKPR303). **Figure S1.** Pearson’s correlation coefficient between two replicates in sterile AKCMS11 (left) and fertile restorer AKPR303 (right). **Figure S2.** Graph representing per base sequence quality. **a)** Sterile AKCMS11 (replicates 1 and 2). **b)** Fertile AKPR303 (replicates 1 and 2).

**Additional File 2: Table S1.** Sequences with significant BLASTX hits against nr protein database. **Table S2.** Sequences with significant BLASTX hits against Virdiplantae database. **Table S3.** Gene Ontology classification of the assembled unigenes. **Table S4.** KEGG classification of *Cajanus cajan* unigenes. **Table S5.** Transcription factors identified in transcriptome of *Cajanus cajan*. **Table S6.** List of differentially expressed genes between sterile AKCMS11 and fertile AKPR303 in pigeon pea. **Table S7.** List of significantly enriched GO terms in down-regulated DEGs. **Table S8.** List of significantly enriched GO terms in up-regulated DEGs. **Table S9.** Summary of KEGG annotations for down and up-regulated DEGs. **Table S10.** TAIR database BLASTX results of pigeon pea, 951 DEGs in MapMan pathway analysis. **Table S11.** OrthoDB homologs search of 3167 pigeon pea DEGs against TAIR database. **Table S12.** List of qRT-PCR primers used in this study.

**Additional file 3: Figure S1.** Gene ontology classification of the assembled unigenes. The Y-axis indicates the number of unigenes and X-axis indicates the GO categories. **Figure S2** Functional classification of KEGG pathways of the assembled unigenes. The KEGG pathways were classified into six functional categories: A- Metabolism; B- Genetic Information Processing; C- Environmental Information Processing; D- Cellular Processes; E- Organismal Systems; F- Human Diseases. The Y-axis represents the KEGG metabolic pathways. The X-axis represents number of unigenes annotated in that particular pathway. **Figure S3** Hierarchical tree graph of over-represented GO terms in the biological process category of down-regulated DEGS. Boxes in the graph represent GO terms labeled according to their GO ID, term definition, and statistical information. Significant terms (adjusted *P* ≤ 0.05) are in color (red, orange, or yellow), while non-significant terms are shown as white boxes. In the diagram, the degree of color saturation of a box is positively correlated with the enrichment level of the term. Solid, dashed, and dotted lines represent two, one, and zero enriched terms at both ends connected by the line, respectively. The rank direction of the graph is set from top to bottom. **Figure S4** Hierarchical tree graph of over-represented GO terms in the biological process category of up-regulated DEGS. Boxes in the graph represent GO terms labeled according to their GO ID, term definition, and statistical information. Significant terms (adjusted *P* ≤ 0.05) are in color (red, orange, or yellow), while non-significant terms are shown as white boxes. In the diagram, the degree of color saturation of a box is positively correlated with the enrichment level of the term. Solid, dashed, and dotted lines represent two, one, and zero enriched terms at both ends connected by the line, respectively. The rank direction of the graph is set from top to bottom.

## Data Availability

The dataset generated and/or analyzed during the current study are available in the NCBI SRA repository (SRX3740150, SRX3740151, SRX3740153, SRX3740152) for the sterile and fertile buds.(https://www.ncbi.nlm.nih.gov/sra/SRX3740153[accn]; https://www.ncbi.nlm.nih.gov/sra/SRX3740152[accn]; https://www.ncbi.nlm.nih.gov/sra/SRX3740151[accn]; https://www.ncbi.nlm.nih.gov/sra/SRX3740150[accn].

## Abbreviations

*ACOS*: *acyl-coA synthetase*
AGPs: Arabinogalactan glycoproteins
*AMS*: *ABORTED MICROSPORES*
AP2: APETALA2
*ARF*: *AUXIN RESPONSIVE FACTOR*
ATP: Adenosine triphosphate
bHLH: Basic helix-loop-helix protein
BLAST: Basic Local Alignment Search Tool
bp: Base pair
bZIP: Basic leucine zipper
C_2_H_2_: Cys2/His2
Cals: Callose synthase
cDNA: Complementary DNA
CMS: Cytoplasmic male-sterility
cox: Cytochrome c oxidase
*CYP703A2*: *CYTOCHROME P450*
*CYP704B1*: *CYTOCHROME P450*
DEGs: Differentially expressed genes
*DYT*: *DYSFUNCTIONAL TAPETUM*
*EMS*: *EXCESS MICROSPOROCYTES*
ERF: Ethylene-responsive factor
FDR: False discovery rate
FLA: Fasciclin-like arabinogalactan
FPKM: Fragments per kilo base per million
GO: Gene Ontology
GST: Glutathione S-transferase
H_2_O_2_: Hydrogen peroxide
KEGG: Kyoto Encyclopedia of Genes and Genomes
*LAP*: Polyketide synthase
LEA: Late embryogenesis abundant
Log_2_ FC: Fold change
LRR-RPK: Leucine-rich repeat receptor protein kinase
MAD: Mitotic spindle assembly checkpoint protein
MDA: Malondialdehyde
*MS*: *MALE STERILITY*
NCBI: National Center for Biotechnology Information
Nr: NCBI non-redundant protein
*NZZ*: *NOZZEL*
O^2^: Superoxide
*orfs*: Open reading frames
PCD: Programmed cell death
PCR: Polymerase chain reaction
PG: Polygalacturonase
PMC: Pollen mother cells
qRT-PCR: Quantitative real-time PCR
*Rf*: Restorer of fertility
RNA-Seq: RNA Sequencing
ROS: Reactive oxygen species
*RPG*: *RUPTURED POLLEN GRAIN*
*SPL*: *SPOROCYTELESS*
TCA: Tricarboxylic acid cycle
TF: Transcription factors
*TPD*: *TAPETUM DETERMINANT*
*UDT*: *UNDEVELOPED TAPETUM*
