# Supplementary figures and images for: Transcriptome profiling of differentially expressed genes in cytoplasmic male-sterile line and its fertility restorer line in pigeon pea (Cajanus cajan L.)
Source: BMC Plant Biol. 2020 Feb 13;20:74. doi: 10.1186/s12870-020-2284-y (PMC7020380; doi:10.1186/s12870-020-2284-y)

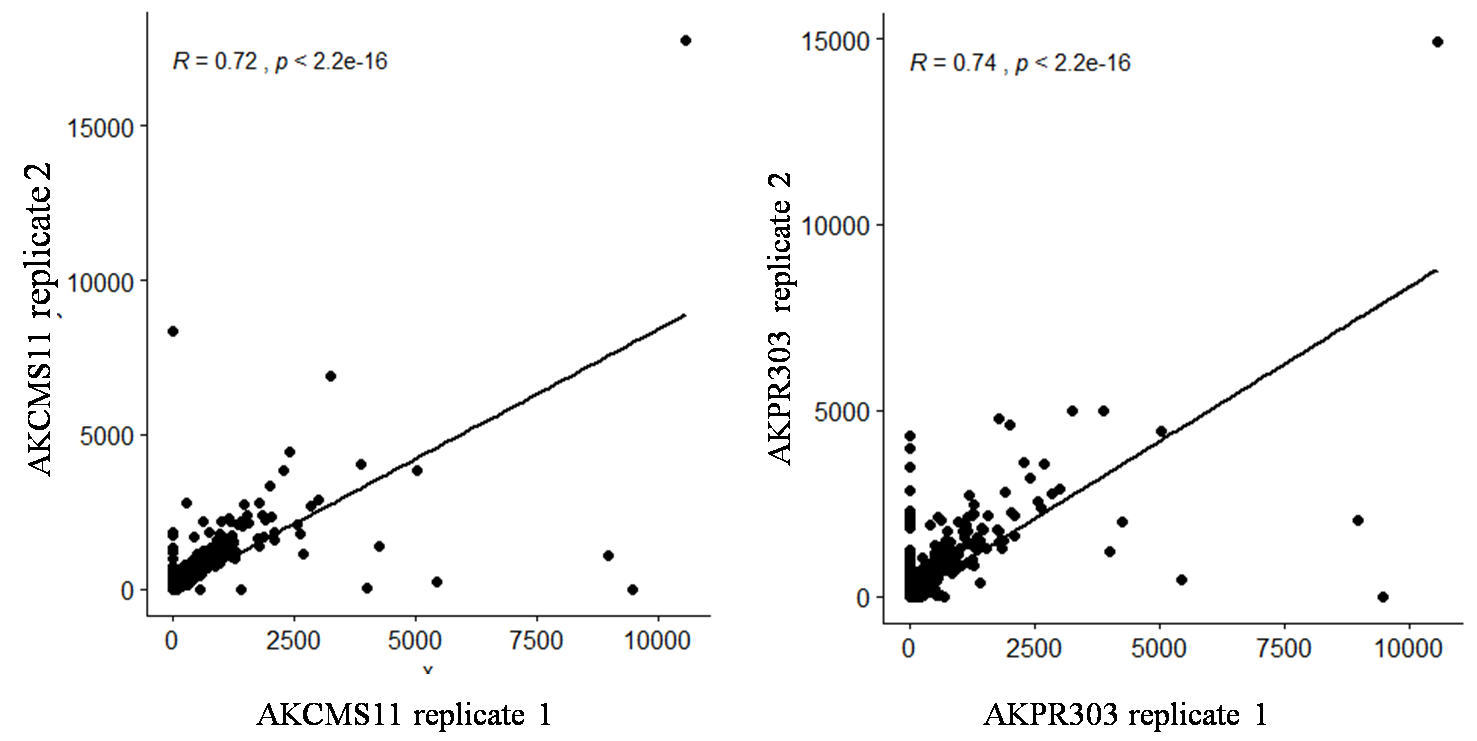

Supplement: Supplementary file 1 — Additional file 1: Table S1. Rcorrector ouput for k-mer content in the raw paired-end data. Table S2. Bowtie2 alignment statistics in sterile (AKCMS11) and fertile restorer (AKPR303). Figure S1. Pearson’s correlation coefficient between two replicates in sterile AKCMS11 (left) and fertile restorer AKPR303 (right). Figure S2. Graph representing per base sequence quality. a) Sterile AKCMS11 (replicates 1 and 2). b) Fertile AKPR303 (replicates 1 and 2). [file 12870_2020_2284_MOESM1_ESM.zip › Additional file 1 Figure S1.tif]

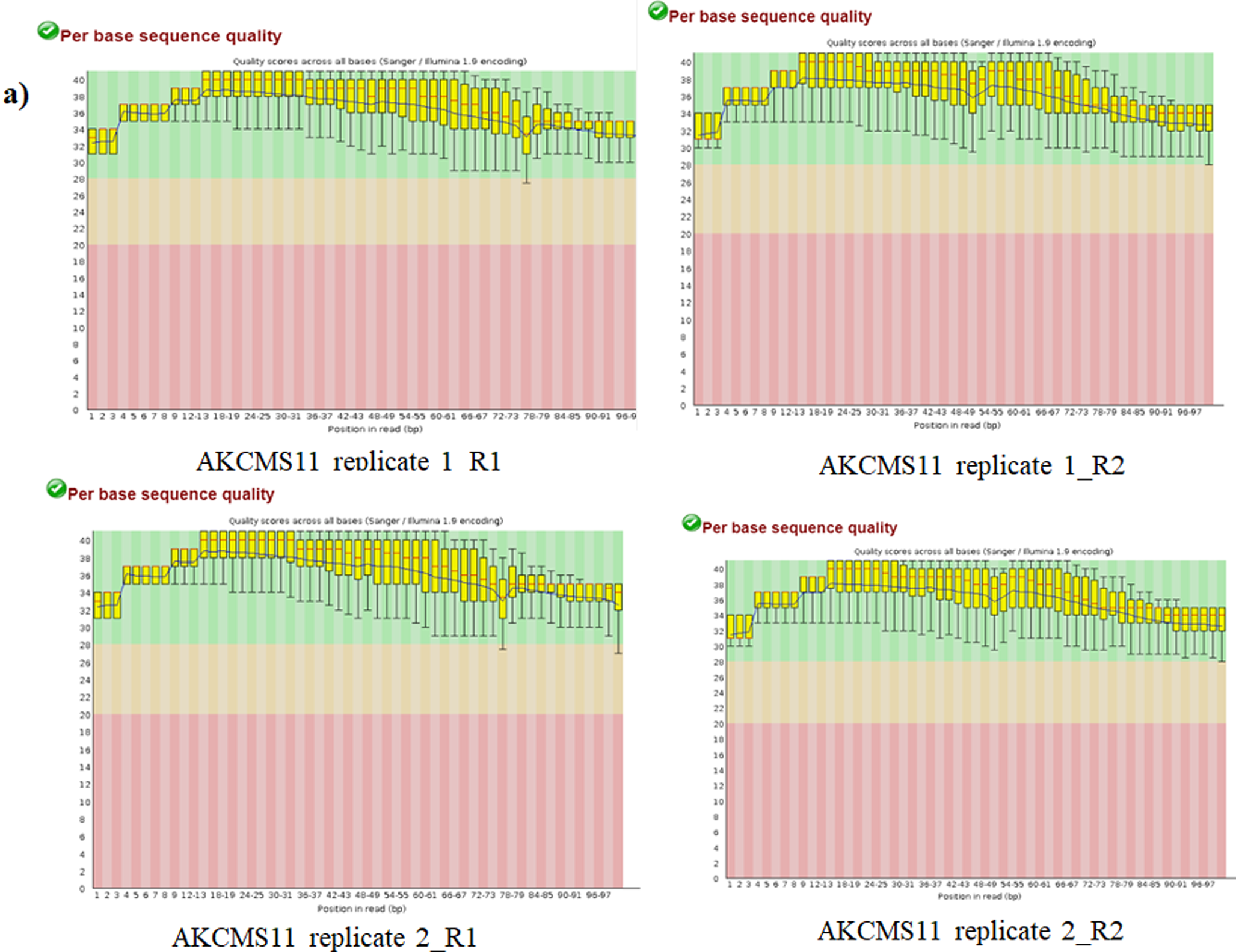

Supplement: Supplementary file 1 — Additional file 1: Table S1. Rcorrector ouput for k-mer content in the raw paired-end data. Table S2. Bowtie2 alignment statistics in sterile (AKCMS11) and fertile restorer (AKPR303). Figure S1. Pearson’s correlation coefficient between two replicates in sterile AKCMS11 (left) and fertile restorer AKPR303 (right). Figure S2. Graph representing per base sequence quality. a) Sterile AKCMS11 (replicates 1 and 2). b) Fertile AKPR303 (replicates 1 and 2). [file 12870_2020_2284_MOESM1_ESM.zip › Additional file 1 Figure S2 (a).tif]

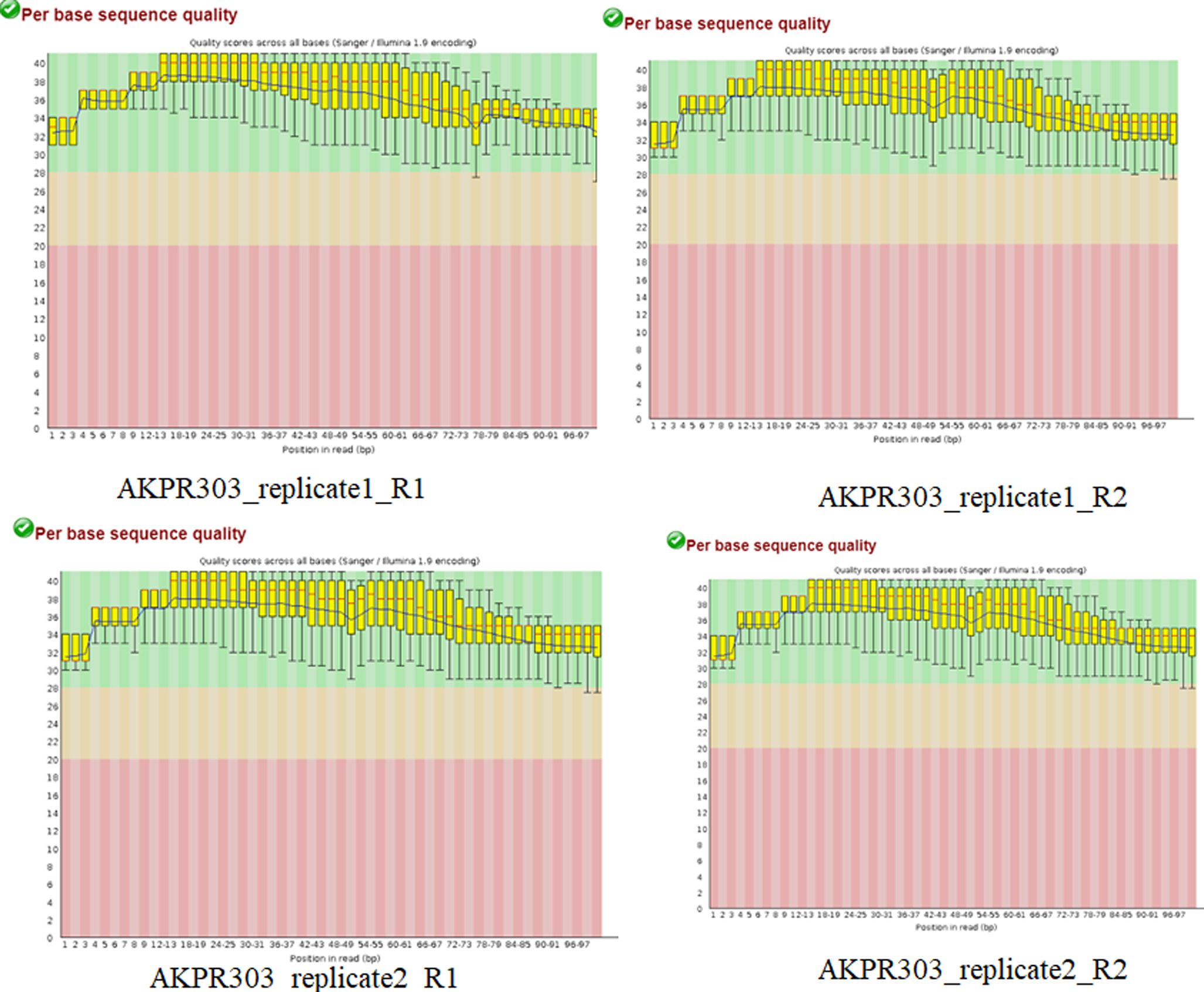

Supplement: Supplementary file 1 — Additional file 1: Table S1. Rcorrector ouput for k-mer content in the raw paired-end data. Table S2. Bowtie2 alignment statistics in sterile (AKCMS11) and fertile restorer (AKPR303). Figure S1. Pearson’s correlation coefficient between two replicates in sterile AKCMS11 (left) and fertile restorer AKPR303 (right). Figure S2. Graph representing per base sequence quality. a) Sterile AKCMS11 (replicates 1 and 2). b) Fertile AKPR303 (replicates 1 and 2). [file 12870_2020_2284_MOESM1_ESM.zip › Additional file 1 Figure S2 (b).tif]

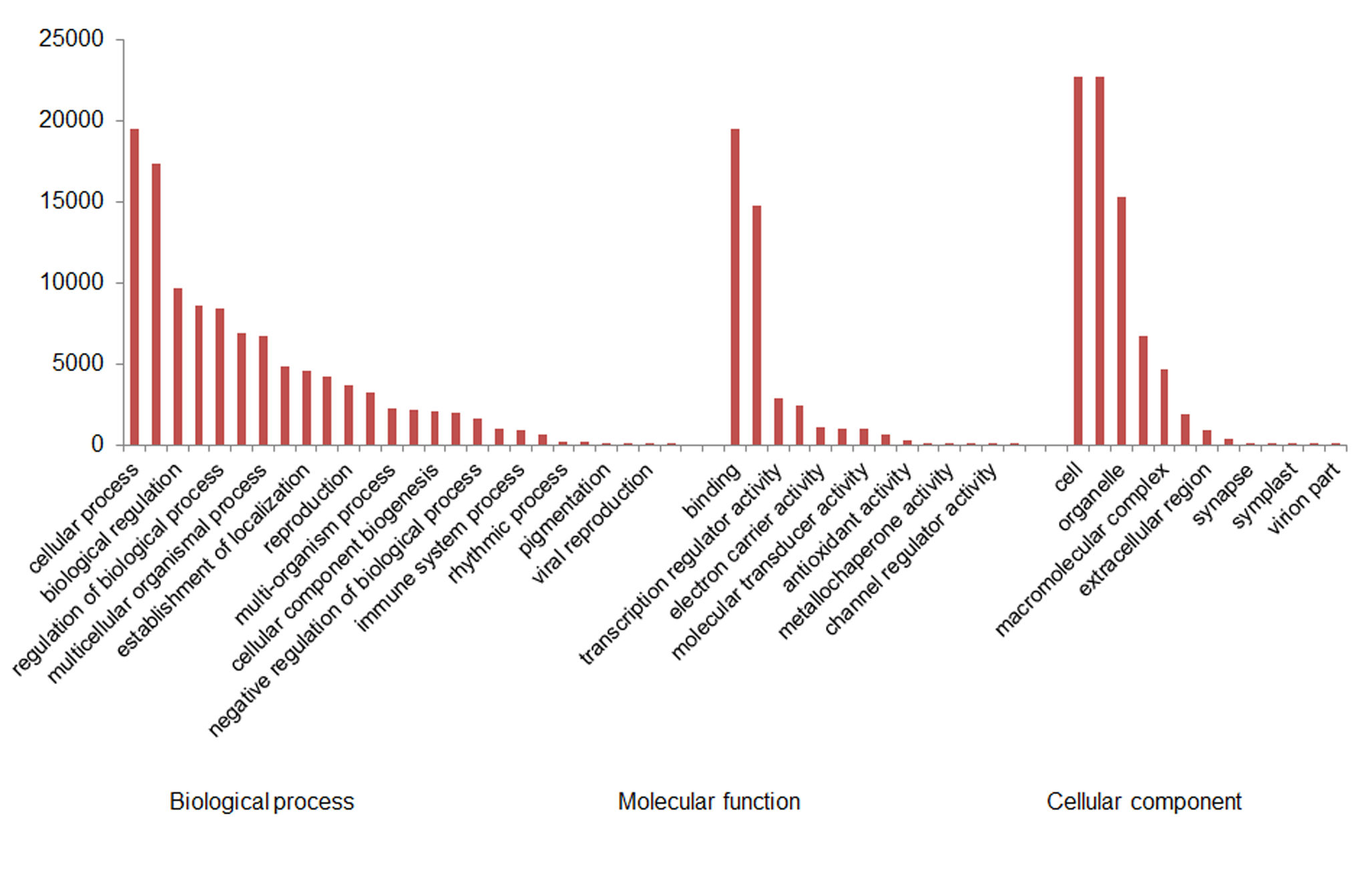

Supplement: Supplementary file 3 — Additional file 3: Figure S1. Gene ontology classification of the assembled unigenes. The Y-axis indicates the number of unigenes and X-axis indicates the GO categories. Figure S2 Functional classification of KEGG pathways of the assembled unigenes. The KEGG pathways were classified into six functional categories: A- Metabolism; B- Genetic Information Processing; C- Environmental Information Processing; D- Cellular Processes; E- Organismal Systems; F- Human Diseases. The Y-axis represents the KEGG metabolic pathways. The X-axis represents number of unigenes annotated in that particular pathway. Figure S3 Hierarchical tree graph of over-represented GO terms in the biological process category of down-regulated DEGS. Boxes in the graph represent GO terms labeled according to their GO ID, term definition, and statistical information. Significant terms (adjusted P ≤ 0.05) are in color (red, orange, or yellow), while non-significant terms are shown as white boxes. In the diagram, the degree of color saturation of a box is positively correlated with the enrichment level of the term. Solid, dashed, and dotted lines represent two, one, and zero enriched terms at both ends connected by the line, respectively. The rank direction of the graph is set from top to bottom. Figure S4 Hierarchical tree graph of over-represented GO terms in the biological process category of up-regulated DEGS. Boxes in the graph represent GO terms labeled according to their GO ID, term definition, and statistical information. Significant terms (adjusted P ≤ 0.05) are in color (red, orange, or yellow), while non-significant terms are shown as white boxes. In the diagram, the degree of color saturation of a box is positively correlated with the enrichment level of the term. Solid, dashed, and dotted lines represent two, one, and zero enriched terms at both ends connected by the line, respectively. The rank direction of the graph is set from top to bottom. [file 12870_2020_2284_MOESM3_ESM.zip › Additional file 3 Figure S1.tif]

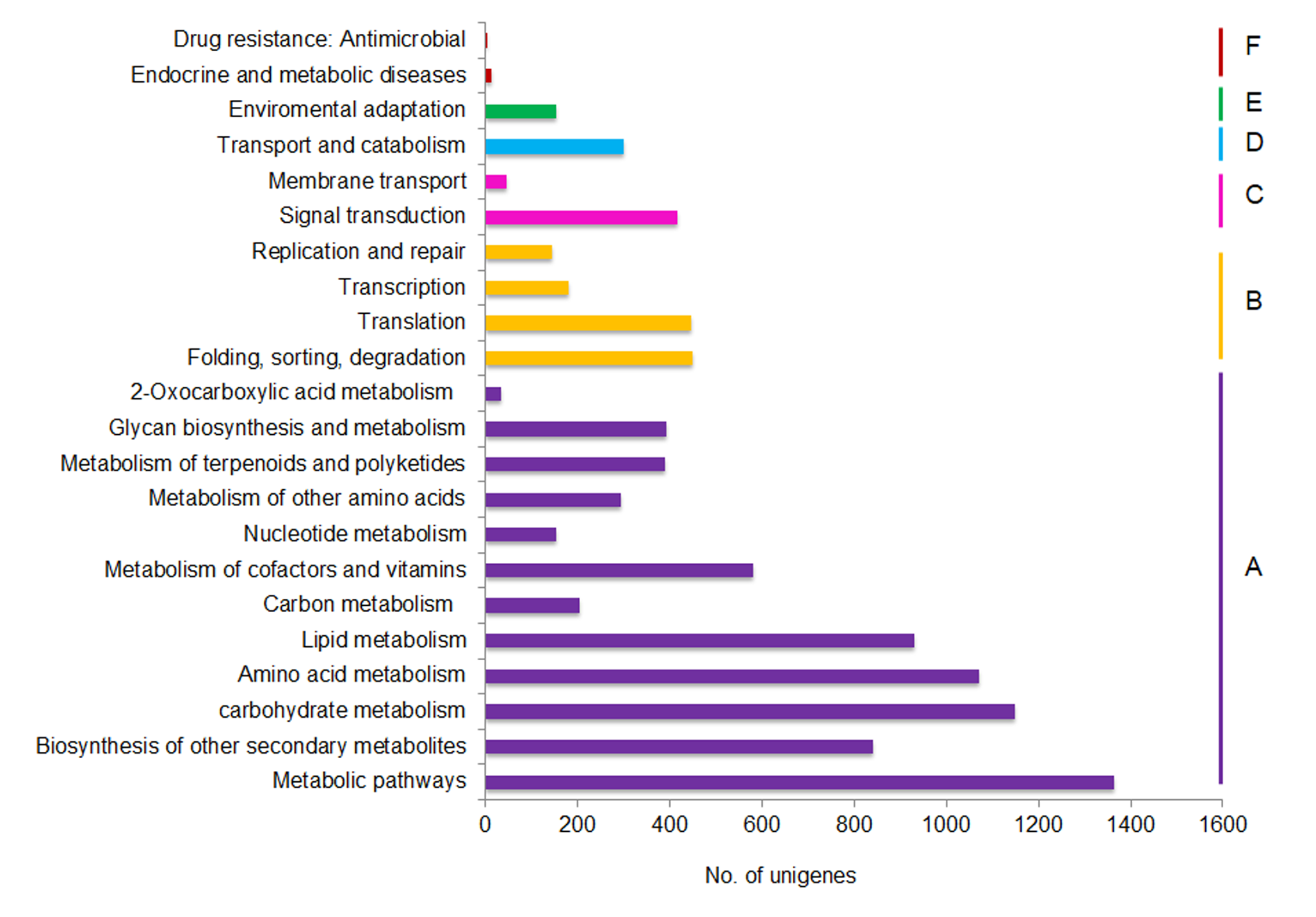

Supplement: Supplementary file 3 — Additional file 3: Figure S1. Gene ontology classification of the assembled unigenes. The Y-axis indicates the number of unigenes and X-axis indicates the GO categories. Figure S2 Functional classification of KEGG pathways of the assembled unigenes. The KEGG pathways were classified into six functional categories: A- Metabolism; B- Genetic Information Processing; C- Environmental Information Processing; D- Cellular Processes; E- Organismal Systems; F- Human Diseases. The Y-axis represents the KEGG metabolic pathways. The X-axis represents number of unigenes annotated in that particular pathway. Figure S3 Hierarchical tree graph of over-represented GO terms in the biological process category of down-regulated DEGS. Boxes in the graph represent GO terms labeled according to their GO ID, term definition, and statistical information. Significant terms (adjusted P ≤ 0.05) are in color (red, orange, or yellow), while non-significant terms are shown as white boxes. In the diagram, the degree of color saturation of a box is positively correlated with the enrichment level of the term. Solid, dashed, and dotted lines represent two, one, and zero enriched terms at both ends connected by the line, respectively. The rank direction of the graph is set from top to bottom. Figure S4 Hierarchical tree graph of over-represented GO terms in the biological process category of up-regulated DEGS. Boxes in the graph represent GO terms labeled according to their GO ID, term definition, and statistical information. Significant terms (adjusted P ≤ 0.05) are in color (red, orange, or yellow), while non-significant terms are shown as white boxes. In the diagram, the degree of color saturation of a box is positively correlated with the enrichment level of the term. Solid, dashed, and dotted lines represent two, one, and zero enriched terms at both ends connected by the line, respectively. The rank direction of the graph is set from top to bottom. [file 12870_2020_2284_MOESM3_ESM.zip › Additional file 3 Figure S2.tif]
